# Transcriptomic Profiling Identifies a Subset of Renal Tumors with Overlapping Features of Clear Cell Papillary Renal Cell Tumor and Renal Cell Carcinoma with Fibromyomatous Stroma

**DOI:** 10.3390/cancers18162713

**Published:** 2026-08-21

**Authors:** Rasmus Jakobsson, Martin Lindström, Yvonne Arvidsson, Iva Johansson, Jonas A. Nilsson, Niels Marcussen, Joakim Karlsson, Martin E. Johansson

**Affiliations:** 1 Department of Laboratory Medicine, Institute of Biomedicine, Sahlgrenska Academy, University of Gothenburg, 405 30 Gothenburg, Sweden; rasmus.jakobsson@gu.se; 2 Department of Surgery and Urology, Skaraborg Hospital, 549 49 Skövde, Sweden; 3 Sahlgrenska Center for Cancer Research, Sahlgrenska Academy, University of Gothenburg, 405 30 Gothenburg, Sweden; 4 Department of Translational Medicine, Lund University, 202 13 Malmö, Sweden; martin.l.lindstrom@skane.se; 5 Department of Clinical Pathology, Laboratory Medicine Skåne, 205 02 Malmö, Sweden; 6 Department of Clinical Pathology, Sahlgrenska University Hospital, 413 45 Gothenburg, Sweden; iva.johansson@vgregion.se; 7 Department of Surgery, Institute of Clinical Sciences, Sahlgrenska Academy, University of Gothenburg, 405 30 Gothenburg, Sweden; 8 Department of Pathology, University Hospital of Southern Denmark, 6200 Aabenraa, Denmark; niels.marcussen@rsyd.dk; 9 Institute of Regional Health Research, University of Southern Denmark, 5230 Odense, Denmark

**Keywords:** renal cell carcinoma, clear cell papillary renal cell tumor, renal cell carcinoma with fibromyomatous stroma

## Abstract

**Background**: Renal cell carcinomas (RCCs) represent neoplasms with variable biological behaviour, some of which remain difficult to classify within the current diagnostic framework. Clear cell papillary renal cell tumor (CCPRCT) is now recognised as an indolent entity, whereas RCC with fibromyomatous stroma (RCCFMS) remains a provisional subtype with partially overlapping morphological features. **Methods**: We analysed a multifocal RCC with clear cell morphology and prominent fibromyomatous stroma via whole-genome and RNA sequencing. The obtained molecular profile was compared with The Cancer Genome Atlas (TCGA) pan-cancer dataset, which includes 885 RCC cases, and histological re-evaluation of 10 identified similar cases was performed. Transcriptional data were mined for potential markers, which were validated in an independent cohort. **Results**: The 10 TCGA cases with similar transcriptomic features were characterised by diploid genomes, absence of recurrent chromosomal alterations, and lack of *VHL* mutations. Reduced *VHL* mRNA expression was observed, with increased methylation at selected CpG sites consistent with possible epigenetic down-regulation. Diagnostic variability was identified during histological re-evaluation of the 10 similar cases by three urological pathologists. Differential expression analysis highlighted cytokeratin 17 (CK17) and collagen 17A1 (COL17A1) as candidate markers. Immunohistochemical evaluation in a small (*n* = 6) independent CCPRCT cohort demonstrated expression of both markers, whereas tissue microarrays from 257 clear cell and 68 papillary RCC cases were found to be negative. **Conclusions**: These findings suggest that a subset of renal tumors with overlapping morphological features of CCPRCT and RCCFMS may share common molecular characteristics. CK17 and COL17A1 emerged as candidate markers for recognising these tumors, although their diagnostic sensitivity and specificity require validation across a broader spectrum of renal neoplasms. These observations are exploratory and hypothesis-generating, and further studies in large, well-characterised cohorts are required to clarify the biological and diagnostic significance of this subgroup.

## Simple Summary

Kidney tumors with clear cell morphology constitute a diverse group, some of which remain difficult to classify. In this study, we performed genomic and transcriptomic sequencing of an index case without a definitive diagnosis and searched for similar cases in the public TCGA database of 33 distinct cancer types, including 885 sequenced kidney tumors. We identified ten kidney tumor cases with extensive similarities regarding genetics, histology, and clinical behaviour. We also identified and validated candidate immunohistochemical markers; however, further validation regarding whether they may aid in recognising this category of tumor with low malignancy potential is warranted. Our findings are exploratory and hypothesis-generating, indicating that further studies of clear cell tumors at the borderline between malignant and benign biology are warranted.

## 1. Introduction

In contrast to many other forms of cancer, the formalised histological classification of renal cell carcinoma (RCC) into distinct kidney tumor categories is relatively recent [1]. While clear cell (CCRCC), papillary (PRCC), and chromophobe (CHRCC) RCC constitute the major RCC subtypes, the increasing application of molecular profiling has led to the recognition of additional entities—as reflected by the 21 distinct RCC subtypes defined in the 2022 World Health Organisation (WHO) classification [2,3]. While this expanded framework has improved diagnostic precision, it has also highlighted challenges in classifying rare or borderline tumors, particularly when diagnostic criteria overlap or remain incompletely defined [4]. This is a significant challenge for the diagnosis of RCCs and tumors with clear cell histology, which are known for their histological diversity within and between cases.

Clear cell papillary renal cell tumor (CCPRCT) and RCC with fibromyomatous stroma (RCCFMS) represent two such diagnostic examples, sharing morphological and immunohistochemical characteristics while differing in classification status and molecular definition.

CCPRCT is a relatively recently defined entity that was included in the WHO Classification in 2016 [5]. In the most recent WHO Classification (2022), it was reclassified and renamed to clear cell papillary renal cell tumor due to its indolent clinical behaviour [2,3]. It is characterised by clear cell histology and low-grade nuclei with an at least focal, apically linearised nuclear position in the cell and a diffuse, so-called “cup-shaped” distribution of carbonic anhydrase 9 (CAIX) expression [2].

Another renal tumor of similar histology was initially described by Michal et al. [6]. This subtype was named renal angiomyomatous tumor (RAT) and is characterised by clear cell morphology and a more distinct fibroleiomyomatous stromal component than CCPRCT [7,8]. Its similarities to CCPRCT led to further studies, and the ensuing debate and analyses resulted in the appreciation that CCPRCT and RAT likely represent the same tumor entity, but with variable morphological features [5].

An additional renal tumor with clear cell morphology—which has been debated as a potential single entity—is RCC with (angio)leiomyomatous stroma, most commonly known as RCC with fibromyomatous stroma (RCCFMS) [9]. This tumor displays clear cell morphology with a prominent, dense spindle-cell stroma resembling fibroblasts and/or smooth muscle cells. It is currently regarded as a provisional entity and is characterised by diffuse CK7 expression and cup-shaped CAIX positivity [2]. Analysing a series of 18 cases, Shah et al. have demonstrated that this category is characterised by somatic mutations in genes implicated in activation of the mTOR signalling cascade: *TSC1*, *TSC2*, *MTOR*, or *ELOC*. *ELOC* encodes for Elongin C, a component of the von Hippel–Lindau tumor suppressor (VHL) E3 ubiquitin ligase complex, and has recently been added to the molecularly defined category in the 2022 WHO classification. Notably, *VHL* was found to be structurally intact in RCCFMS [10]. Clinically, RCCFMS is typically associated with indolent behaviour; however, it is still classified as a low-grade malignant renal neoplasm due to occasional reports of metastasis, most often in the context of tuberous sclerosis syndrome [11]. Other reports may also represent tumors within the broader fibromyomatous spectrum, including *ELOC*-mutated RCC [12].

The distinction between CCPRCT and RCCFMS in routine practice relies primarily on morphological criteria such as nuclear positioning and the extent and composition of stromal elements [13,14]. However, these features may overlap and stromal components are not entirely specific, as similar findings can be observed in other RCC subtypes. Alterations in *TSC1, TSC2*, and *MTOR* in RCCFMS are regarded as bona fide driver events, leading to constitutive mTOR activation. The morphologic spectrum most probably encompasses molecularly heterogeneous subgroups; however, there are limited publicly available molecular datasets for recently recognised RCC subtypes. In addition, the TCGA RCC cohort consists of tumors diagnosed more than two decades ago, when clear cell, papillary, and chromophobe RCC represented the predominant diagnostic categories. It is well recognised that a proportion of these tumors would now be reclassified according to the current WHO classification. Therefore, we compared the index case with the complete TCGA RCC cohort, rather than the original diagnosis, to guide case identification.

The TCGA dataset comprises an exhaustive molecular analysis of 33 major tumor types, including the three main RCC groups CCRCC, PRCC, and chromophobe RCC (CHRCC) [15,16,17], and the large number of molecularly defined cases makes it a valuable source for taxonomic studies. It is recognised that the TCGA data may contain less common entities that were previously attributed to the three major RCC types, thus warranting reclassification [18].

To explore these lesser-studied clear cell entities and their potential relationships, we conducted an index-case-driven exploratory analysis and analysed a case fulfilling the histological criteria of an RCCFMS via whole-genome and transcriptomic profiling. We then performed an exploratory transcriptome-wide comparison with TCGA data to identify tumors with similar molecular characteristics. Through this approach, we found data suggesting that a subset of renal tumors with fibromyomatous stroma shares some molecular and immunohistochemical features with CCPRCT, and subsequently identified candidate markers that may aid in their recognition.

## 2. Materials and Methods

### 2.1. Case Selection

We identified a 50-year-old male patient with hypertension who was incidentally diagnosed with multiple tumors bilaterally in the kidneys, with a largest tumor measuring 35 mm in diameter. Biopsies had been obtained from some of the tumors as part of the diagnostic work-up; however, the morphology and IHC results were not typical for CCRCC. First, the cup-shaped distribution of the CAIX signal was regarded as a problematic diagnostic feature indicating CCPRCT. On the other hand, no apical positioning of the nuclei was observed in the limited material available for evaluation, thus precluding an unequivocal benign diagnosis. The patient underwent resection of the tumors in their left kidney. In total, six tumors were identified and histologically evaluated (T1–T6). The histology was similar in all tumors, and two (T3 and T4) were randomly selected for whole-genome sequencing (WGS). With additional material available for evaluation, the tumor was considered to exhibit morphological features of RCCFMS, with CCPRCT representing the closest established subtype. We obtained consent from the patient and approval from the Regional Ethical Review Board of Gothenburg for the use of clinical materials for research purposes, including genetic analysis (Dnr: 2019-00905).

### 2.2. Immunohistochemistry

Tissues from the two tumors were formalin-fixed, paraffin-embedded, and subjected to antigen retrieval using EnVision FLEX Target Retrieval Solution (high pH) (Dako PT-Link, Agilent Technologies, Santa Clara, CA, USA). Immunohistochemical staining was performed in a Dako Autostainer Link using EnVision FLEX, according to the manufacturer’s instructions (DakoCytomation, Agilent Technologies, Santa Clara, CA, USA). Histopathological evaluation was performed using haematoxylin and eosin (H&E)-stained sections and immunohistochemical (IHC) staining for CAIX, cytokeratin 7 (CK7/KRT7), cluster of differentiation 10 (CD10), vimentin, epithelial antibody cocktail 1-3 (AE1-3), epithelial membrane antigen (EMA), and E-cadherin (ECAD). Additional IHC staining was performed using antibodies against Collagen Type XVII Alpha 1 Chain (COL17A1, 1:250, no. HPA043673, Atlas Antibodies, Stockholm, Sweden)), cytokeratin 17 (CK17/KRT17, ready to use, no. IR620, Agilent Technologies, Santa Clara, CA, USA), phospho-Mechanistic Target Of Rapamycin Kinase (p-MTOR, 1:25, no. 2976, Cell signalling technology, Danvers, MA, USA), phospho-S6 Kinase 1 (pS6K1, 1:500, no. ab60948, Abcam, Cambridge, UK), VHL (1:1000, no. sc-135657, Santa Cruz Biotechnology, Dallas, TX, USA), and Tuberous Sclerosis Complex 2 (TSC2, 1:100, no. HPA030409, Atlas Antibodies, Stockholm, Sweden). More information regarding the antibodies and staining patterns is provided in Supplementary Table S1. The biopsies were reviewed by a board-certified surgical pathologist (M.J.).

### 2.3. Whole-Genome Sequencing and RNA Sequencing

Supplementary Figure S2 shows the analytical workflow for the index case analysis and identification of similar cases. DNA and RNA were isolated from snap-frozen biopsies from two tumors in the left kidney using the allprep DNA/RNA Mini Kit (Qiagen, Hilden, Germany), according to the manufacturer’s protocol. The two tumors originated from the same patient and were analysed separately to assess intrapatient heterogeneity and subclonal variation; they were not considered independent biological samples. Blood was collected from the patient, from which DNA was extracted using the DNeasy Blood & Tissue Kit (Qiagen, Hilden, Germany) and considered representative of normal tissue. WGS libraries were constructed from the two tumors using the TruSeq PCR free kit (Illumina, San Diego, CA, USA) and sequenced on an Illumina Novaseq, (Illumina, San Diego, CA, USA) 6000 using 150 bp paired-end reads to an average depth of 893 million reads (average coverage: 75X). Transcriptome libraries were constructed from the two tumors using the TruSeq Stranded Total RNA Sample Preparation Kit with Ribo-Zero Gold (Illumina, San Diego, CA, USA) and sequenced on a Nextseq500 sequencing system, (Illumina, San Diego, CA, USA) using 2 × 75 bp reads to an average depth of 44 million reads. WGS and RNA sequencing were performed at the Genomics Core Facility at the Sahlgrenska Academy, University of Gothenburg.

### 2.4. RNA-Seq Processing and Normalisation

To facilitate direct comparison, RNA-seq data from the index case and TCGA were processed using the same bioinformatic pipeline for alignment and gene expression quantification. Raw RNA-seq reads were aligned to the UCSC hg19 human genome assembly, excluding alternative haplotype regions, with hisat 0.1.6-beta (parameters: *-*-no-mixed --no-discordant --no-unal --known-splicesite-infile) and converted to BAM format using samtools (v. 0.1.19) [19]. RNA-seq data from TCGA for 9583 tumors from 32 cancer types were downloaded from the cgHub repository on 18 December 2015 and aligned in the same manner. Gene read counts were derived using htseq-count (parameters: -m intersection-strict -s no) [20]. For TCGA data, reads per kilobase per million (RPKM) normalised values were calculated, considering the maximum mature transcript length of each gene and using robust size factors as previously described for the DESeq method. Reads from our samples were normalised using the same method, except that standard read-depth-based size factors were used for RPKM normalisation.

### 2.5. Gene-Expression-Based Classification

To classify a sample using the *k*-nearest neighbour approach, Spearman correlation coefficients with respect to all coding genes were first calculated between the sample of interest and all samples in the TCGA pan-cancer dataset (*cor.test* in R, with the parameter method = “spearman”). A prediction was then made using the majority vote of the top *k* most strongly correlated samples. In the case of ties, the value of *k* was decreased by one until a majority vote was achieved. A value of *k* = 6 was used, which was previously found to be optimal using leave-one-out cross-validation [21]. The k-nearest neighbour classifier (*k* = 6) was used as previously described to support transcriptomic classification, while visualisation of sample relationships was achieved using *t*-SNE. For *t*-distributed stochastic neighbour embedding (*t*-SNE) analysis, log_2_-transformed (pseudo count of 1 added) expression values of all coding genes were used as inputs for the *Rtsne* function from the “Rtsne” R package (v. 0.15) [22]. No predefined correlation threshold was used.

### 2.6. Differential Expression and Gene Set Enrichment Analysis

Wilcoxon rank-sum tests were used to compare the differential expression between primary case samples or similar RCC samples identified in TCGA and the remaining TCGA CCRCC (KIRC) and PRCC (KIRP) samples (*wilcox.test* in R). Adjusted *p*-values (Benjamini–Hochberg correction) < 0.1 were considered significant. Log_2_ fold changes were calculated as log_2_([mean group A + 1]/[mean group B + 1]). To determine over/under-expressed genes, the intersection of genes that were significant and showed the same direction of fold change was considered. For comparative pathway analysis, single-sample gene set enrichment analysis was performed using the GSVA R package (v. 1.36.3), with the function *gsva* (method = “ssgsea”, kcdf = “Poisson”) and Hallmark pathways obtained from MSigDB (*h.all.v7.1.symbols.gmt*) [23]. Differences in normalised enrichment scores across the sample groups of interest were assessed using two-tailed *t*-tests (*t.test* in R), and the resulting *p*-values were adjusted using the Benjamini–Hochberg correction. A False Discovery Rate (FDR value) < 0.05 was considered significant. Gene set enrichment among genes that differed in expression between the two main case tumors was assessed based on log_2_ fold changes using the fgsea R package (v. 1.12.0) with the same set of reference pathways (parameters: minSize = 0, maxSize = 10,000, nperm = 10^7^) [24].

### 2.7. DNA-Seq Processing

Raw WGS reads were aligned to the 1000 Genomes version of the GRCh37 human reference genome (human_g1k_v37_decoy.fasta), with decoy sequences included, using bwa (v. 0.7.17-r1188; parameters: mem -t 40 -M -R) [25]. Samples sequenced across multiple lanes were merged using SAMtools (v. 1.10). Duplicate reads were marked using GATK (v. 4.1.3.0) module MarkDuplicates [26], and base quality scores were recalibrated using the GATK commands BaseRecalibrator and ApplyBQSR in two passes, with the same reference genome used for alignment and with population variant resources obtained from the GATK resource bundle (*dbSNP_138.b37.vcf*,*1000G_phase1.indels.b37.vcf*,*Mills_and_1000G_gold_standard.indels.b37.vcf*).

### 2.8. DNA Copy Number Analysis

Matched tumor and normal WGS BAM files were analysed using CNVkit (v. 0.9.6) via the *batch* command (with the parameters -m --male-reference), and segmented using the *export seg* command in CNVkit (v. 0.9.6).

### 2.9. Mutation Calling

Variant calling using WGS of paired tumor and normal samples was performed using Mutect 2 (GATK v. 4.1.3.0) with the following parameters: --genotype-germline-sites true, --af-of-alleles-not-in-resource 0.0000025, --disable-read-filter MateOnSameContigOrNoMappedMateReadFilter, and --germline-resource [27]. The gnomAD database was used as a germline resource, as provided in the GATK resource bundle (*af-only-gnomad.raw.sites.b37.vcf*) [28]. The quality of the resulting raw variant calls was assessed with FilterMutectCalls (GATK), using the same reference genome as for alignment. Further annotation was performed using maftools (v. 1.6.17), which uses VEP (v. 98.2), utilising a version of the ExAC germline variant database with TCGA samples excluded (*ftp://ftp.broadinstitute.org:/pub/ExAC_release/release0.3.1/subsets/ExAC_nonTCGA.r0.3.1.sites.vep.vcf.gz*). The following variants were then removed: those with population frequency ≥ 0.001 in gnomAD or ExAC, those present in either the dbSNP or ESP databases but not COSMIC, and those not labelled as PASS by FilterMutectCalls (v. 4.1.3.0). Prediction of impact for protein sequence variant queries was performed using PolyPhen and SIFT [29,30].

### 2.10. Analysis of Data from the TCGA Database

A complete description of the workflow for establishing the TCGA RCC database, including tumor identification, sequencing procedures, and methylation arrays, has previously been described [31]. In addition to sequencing data, diagnostic H&E slides, clinical records, and pathology reports are available from the National Cancer Institute Genome Data Commons (https://gdc.cancer.gov/). An easy-access overview of histology and pathology reports is also provided by Emory University (Atlanta, GA, USA) (https://cancer.digitalslidearchive.org) [32]. The analysis was performed using the complete TCGA RCC cohort (*n* = 885). Transcriptomic similarity between T3, T4, and all TCGA RCC samples was assessed using genome-wide Spearman correlations across coding genes, and the relationships between samples were visualised via unsupervised t-SNE analysis. The ten TCGA tumors included in the subsequent analyses comprised all cases forming the distinct transcriptomic cluster together with the index case. No predefined correlation threshold was applied and the comparison was intentionally performed against the complete TCGA RCC cohort, rather than selected histological subtypes, to minimise classification bias and allow transcriptomic similarity to drive case identification. Clinical and histopathological features were evaluated only after transcriptomic identification and were therefore not used as selection criteria. Three experienced urological pathologists (M.L., N.M., and M.J.) from different institutions independently reviewed the digital H&E slides without a formal consensus or adjudication process. The purpose of the review was to assess the degree of morphological agreement between the observers using current diagnostic criteria.

### 2.11. Verification of Marker Utility

To evaluate the utility of the potential markers CK17 and COL17A1 in identifying CCPRCT and RCCFMS, we stained tissue microarrays (TMAs) of CCRCC (*n* = 257 cases) and PRCC (*n* = 68 cases), which served as negative verification of the markers. The TMAs were constructed using two representative punch biopsies from surgically resected RCC tumors at a single centre (Malmö University Hospital, Malmö, Sweden). For positive verification, tissue from the index case was used in addition to whole-tissue sections from five consecutive, recently diagnosed CCPRCT cases. Histological evaluation was performed by three independent assessors (M.J., R.J., and M.L.).

### 2.12. Histological Assessment of 10 Identified Similar Tumors from the TCGA Data Repository

Digitalised histological images from the similar cases identified in the TCGA dataset were downloaded and independently diagnosed by three pathologists specialised in urological pathology working at three different university hospitals. Diagnostic criteria were derived from the 2022 edition of the WHO manual.

## 3. Results

### 3.1. Histopathological Evaluation and Marker Assessment of the Index Case

**Figure 1:**
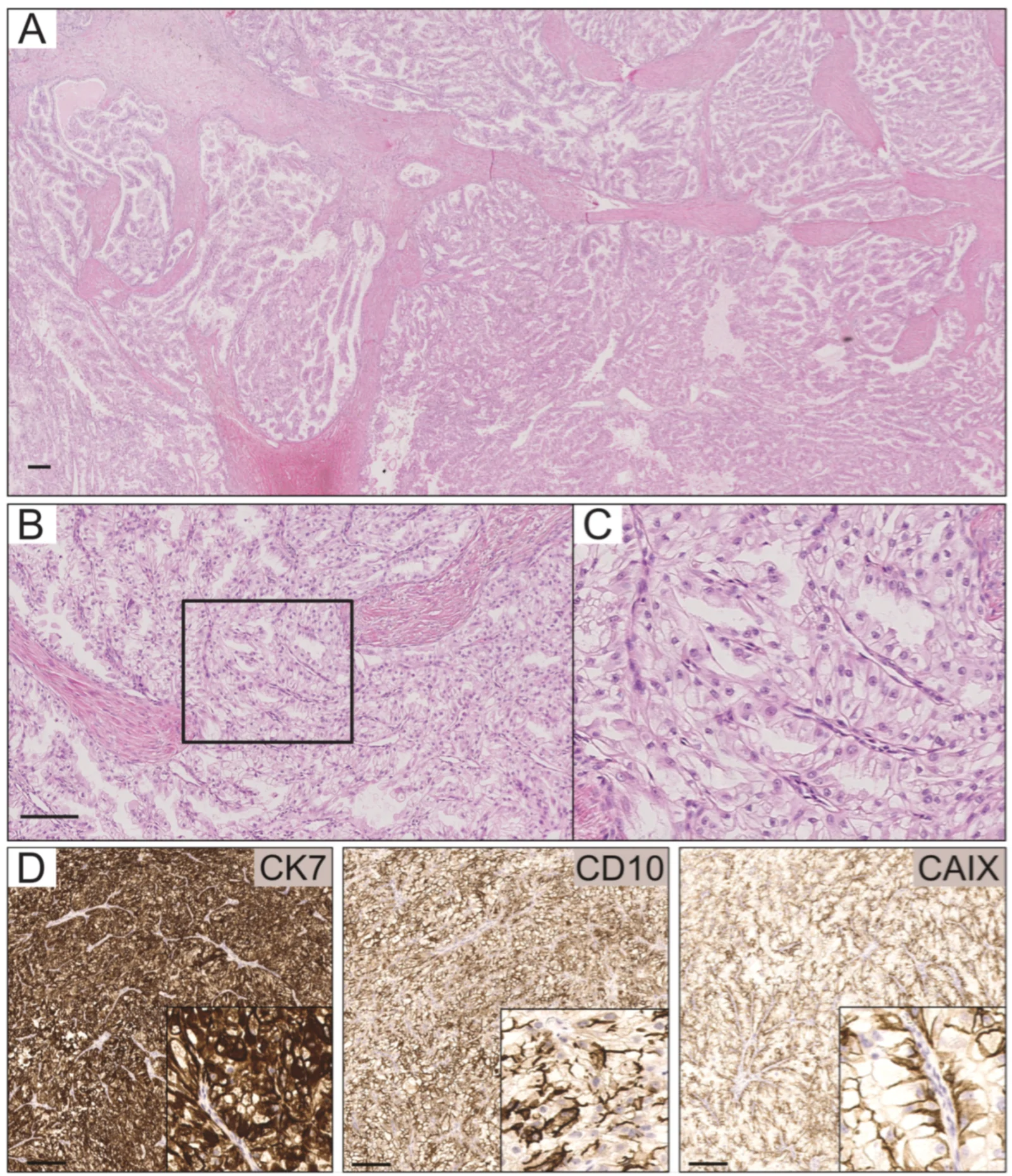
Histological features of the index case: (**A**) Overview of the histology, showing the dense fibromatous strands separating the clear cell tumor component. The tumor cells are arranged in papillary and tubular/acinar patterns. (**B**,**C**) Higher magnification, showing details of the histology and mainly basally oriented nuclei. (**D**) Cytokeratin 7 (KRT7/CK7) and cluster of differentiation 10 (CD10) show diffuse, high expression in the tumor component. Carbonic anhydrase IX (CAIX) with basolateral (cup-shaped) appearance. Boxed areas show histology at higher magnification. Scale bars = 200 μm. Magnified square in Figure (**D**) = 200 × 200 μm. HE, haematoxylin/eosin staining.

Standard histopathological analysis using H&E staining was performed on four tumors excised from the left kidney of the patient. Microscopically clear or slightly eosinophilic cells were arranged in branching papillary and tubular patterns separated by a focally prominent dense fibromuscular stroma. The nuclei of the tumor cells were mainly localised basally, and no areas with apically localised nuclei were present. IHC revealed diffuse CK7 positivity and basolateral (“cup-shaped”) CAIX positivity. Diffuse CD10 positivity was also noted (Figure 1A–D). The tumors were considered to show morphological features of RCCFMS, with CCPRCT as the closest differential diagnosis. Additional clinically relevant markers were also tested (Supplementary Table S1).

### 3.2. Expression Analysis and Identification of Similar Tumors in the TCGA Dataset

**Figure 2:**
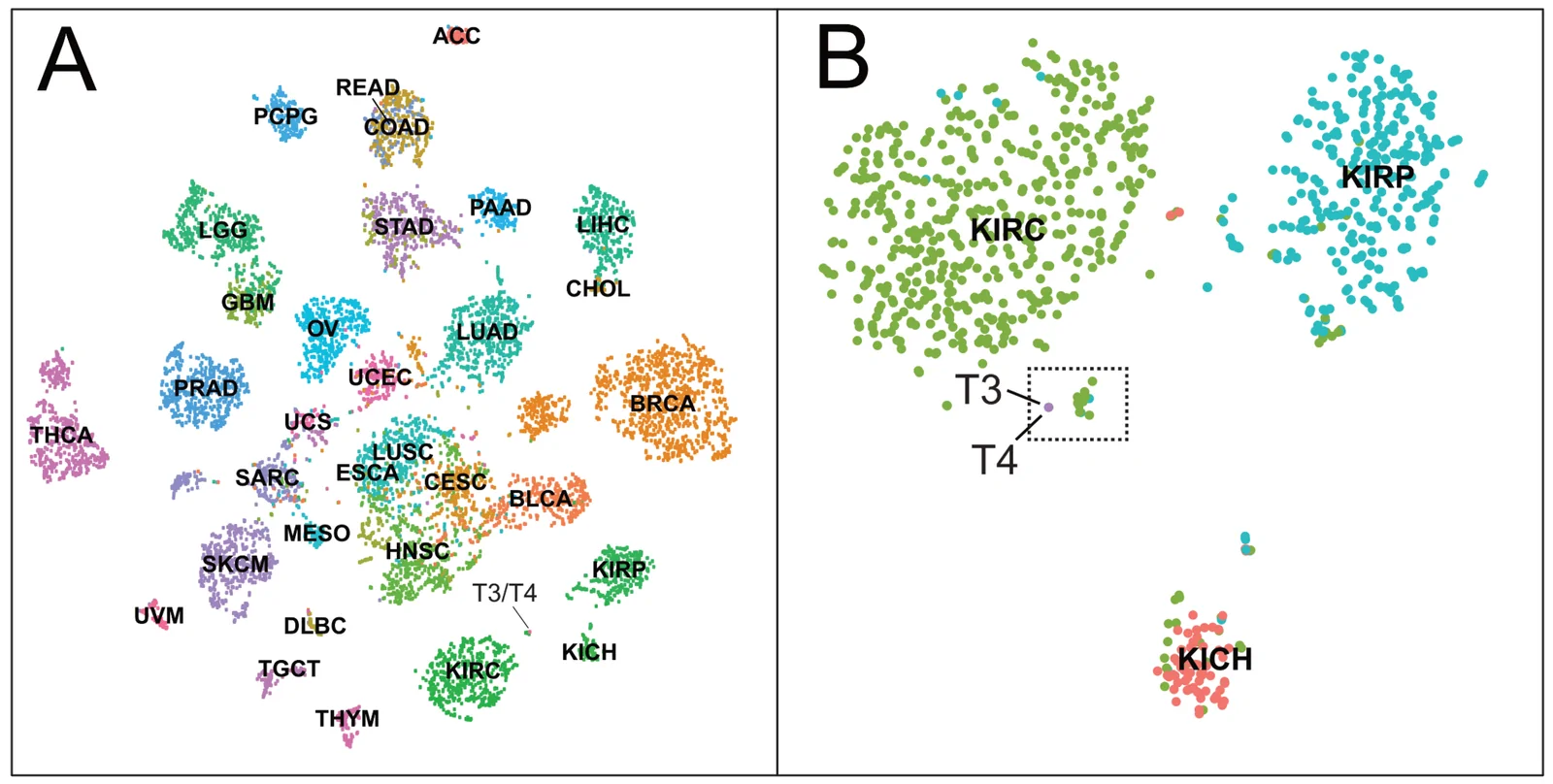
Unsupervised *t*-SNE analyses in two dimensions of RNA-seq data from the index case annotating T3 and T4 to The Cancer Genome Atlas (TCGA): (**A**) Comparative analysis using the entire TCGA dataset of 32 cancer types (9583 cases). Samples are coloured according to known cancer type, forming clusters in the *t*-SNE space. (**B**) Exploration of the TCGA RCC samples relative to the index case tumors. A distinct sub-cluster with 10 similar RCC samples adjacent to the index case tumor samples T3 and T4 was identified. RCC, renal cell carcinoma; KIRC, clear cell RCC; KIRP, papillary RCC; KICH, chromophobe RCC. See Table S6 for complete abbreviation list for TCGA.

Fresh frozen samples from the spatially distinct T3 and T4 were subjected to whole-genome transcriptome sequencing to assess intrapatient heterogeneity and subclonal variation; these analyses should therefore be regarded as exploratory, rather than representing independent biological observations. The tumors were compared with 885 RCC tumors from the TCGA database (530 CCRCC (KIRC), 289 PRCC (KIRP), 66 CHRCC (KICH)). Unsupervised *t*-SNE analysis of the RNA-seq data revealed a transcriptomic world map of the 32 available cancer types in TCGA (Figure 2A). T3 and T4 were separately analysed for their similarities with the RCC tumors, revealing that the index case tumors clustered with a specific subset of TCGA RCC tumors and formed a distinct separate cluster, all of which were included in the subsequent analysis (Figure 2B). The unique identification numbers are provided in Supplementary Table S4. When T3 and T4 were included in the same analysis, the distance to the other tumors increased due to the similarities between the two patient samples; however, clustering remained evident.

### 3.3. Copy Number Profiles and VHL Expression

**Figure 3:**
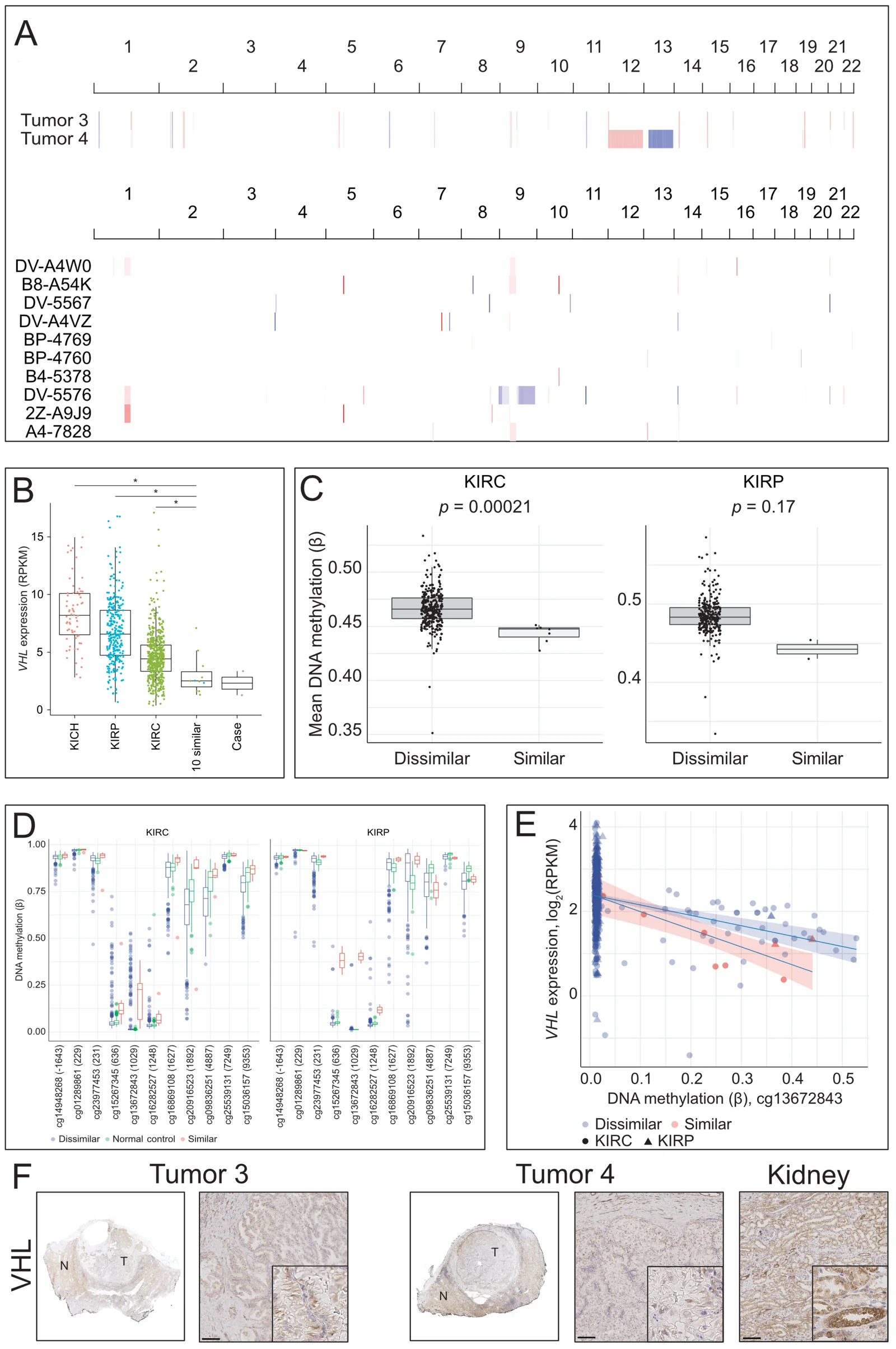
Genomic and histological analysis suggests strong similarities within the identified cluster: (**A**) Somatic copy number profiles of index case T3 and T4 and the identified 10 similar tumors. No major genomic alterations are present, such as chromosomal loss or rearrangements. Blue markings indicate copy number gain, red indicate copy number loss, with increasing colour intensity representing a greater magnitude of copy number alteration. (**B**) Transcriptomic analysis of *VHL* expression. Stars indicate significantly (*p* < 0.05) lower average expression compared to KICH, KIRP and KIRC of the identified sub-cluster. (**C**) Mean DNA methylation of the 10 transcriptomically similar cases compared to KIRC and KIRP cases. The 10 similar cases exhibited significantly lower methylation levels than the KIRC cases. (**D**) Methylation levels at individual CpG (cytosine–phosphate–guanine) sites within VHL, measured using the Illumina Methylation Array. Differences in mean methylation between the 10 identified cases and KIRC and KIRP demonstrate several CpG probes with divergent methylation patterns between the groups. (**E**) *VHL* expression correlated against DNA methylation of cg13672843 located at exon 1. A significant decline in *VHL* expression is noted with increasing methylation. (**F**) Immunohistochemistry of our case, showing lower VHL protein expression compared to normal kidney tissue. Scale bar = 200 μm. Magnified square = 200 × 200 μm. *VHL*, von Hippel–Lindau tumor suppressor gene; KIRC, clear cell RCC; KIRP, papillary RCC; KICH, chromophobe RCC; N, normal tissue; T, tumor tissue.

The somatic copy number profiles indicated largely diploid genomes in the index case tumors and the ten most similar TCGA tumors, further supporting their similarity. Sub-clonal variations in T4 included gain of chromosome 12 and loss of chromosome 13. Chromosome 3p was preserved in all 10 similar tumors and the index case tumors (Figure 3A). No genomic alterations in *VHL* were detected in T3 or T4 and, when analysing the 10 similar cases, only one harboured a genetic mutation in *VHL*. Expression analysis of *VHL* showed significantly lower expression in the index case tumors and the 10 similar tumors (Figure 3B). In addition, in the sub-cluster, the degree of methylation was generally low, except for *VHL*, for which methylation levels were higher in the group. Specifically, several important CpG probes exhibited significantly increased methylation, consistent with the notion that increased methylation may contribute to reduced *VHL* expression (Figure 3C,D); in particular, increased methylation of the probe cg13672843 was associated with lower *VHL* expression in the sub-cluster, when compared to other RCC tumors (Figure 3E). IHC of the case tumors revealed lower VHL protein expression in comparison with normal kidney tissue (Figure 3F).

### 3.4. Somatic Mutations and Differential Expression

**Figure 4:**
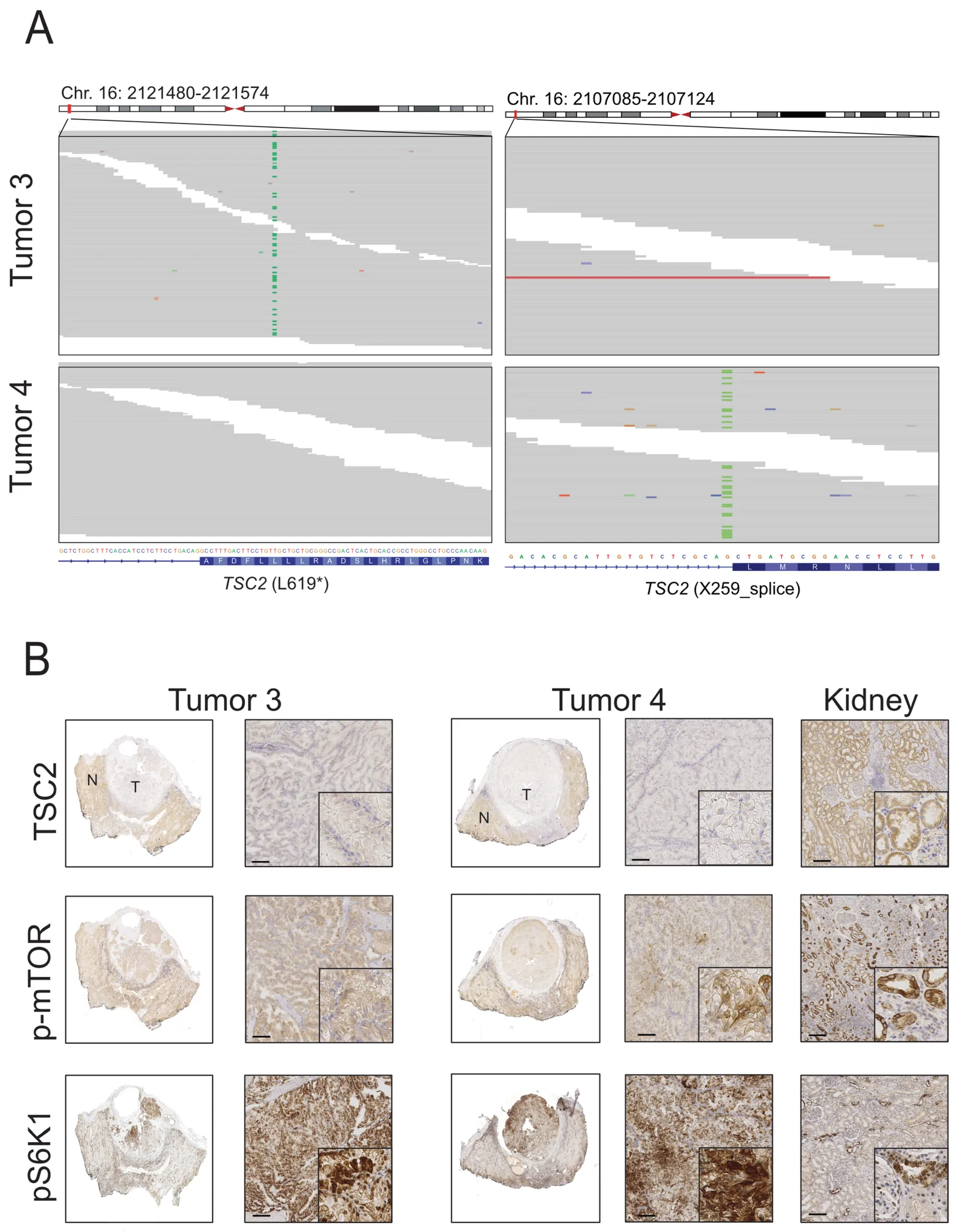
Analysis of mutational status of the index case: (**A**) Subclonal mutations of *TSC2* for tumors 3 and 4. T3 harbours a nonsense mutation (stop-gain) at L619, and T4 presents a splice site mutation (rs45454192). Green indicates sequence variants in reads at specific bases. The red line in T3 represents a deletion observed in a single read and was interpreted as an artefact. (**B**) Immunohistochemistry for TSC2 (low expression), p-mTOR (generally low expression, with higher expression in demarcated areas), and pS6K1 (high expression) suggests activation of the mTOR pathway in index case. Scale bar = 200 μm. Magnified square = 200 × 200 μm. TSC2, Tuberous Sclerosis Complex 2; p-mTOR, phospho-Mechanistic Target of Rapamycin Kinase; pS6K1, phospho-S6 Kinase 1; N, normal tissue; T, tumor.

One out of the identified 10 similar cases had a mutation in *MTOR*. No other mutations were identified for *TSC1*, *TSC2*, *ELOC,* or *MTOR*. In the index case, a nonsense mutation (stop-gain) at L619 of *TSC2* was observed in T3. T4 exhibited a splice site mutation (rs45454192) in *TSC2*, where 33% of the reads did not splice correctly for *TSC2* (Figure 4A). A germline TSC2 variant, NM_000548.5:c.674T>G (p.Val225Gly), was identified in normal and tumor samples. This variant is absent from the population databases dbSNP and gnomAD. In silico prediction tools (SIFT and PolyPhen-2) predicted the variant to be deleterious; however, computational predictions alone are insufficient to establish pathogenicity, and the clinical significance of the variant remains uncertain. Complementary IHC for TSC2 (low expression), p-mTOR (generally low expression, with higher expression in demarcated areas), and pS6K1 (high expression) indicated that loss of function of TSC2 resulted in activation of the mTOR pathway (Figure 4B). Serial sections of the tumors displayed colocalised IHC staining for p-mTOR and pS6K1, while expression analysis of T3 and T4 highlighted low *TSC2* expression (Supplementary Figure S3). Gene set enrichment analysis of gene expression in T3 revealed higher levels of genes involved in mTOR complex 1 signalling relative to T4, and gene set enrichment analysis of the index case and the ten similar cases with respect to MSigDB Hallmark pathways showed higher-than-average hypoxia and transforming growth factor beta (TGF-β) signalling and lower activity of pathways related to the handling of reactive oxygen species (Supplementary Figure S1).

### 3.5. Clinical and Histopathological Data from the 10 Similar Cases

The clinical data for the 10 similar cases in the TCGA data repository and the index case are summarised in Table 1. A comprehensive histological assessment was also performed for all similar cases based on digitalised H/E slides. Immunohistochemistry slides were not available for evaluation. Re-evaluation of the histology by the three independent urological pathologists revealed substantial interobserver variability (Table 2). It should be noted that a formal consensus review was deliberately not undertaken, and the individual assessments are presented to illustrate the diagnostic variability observed between experienced observers. Most of the 10 similar cases displayed distinct features of CCPRCT, mainly with clear cell morphology and areas showing an apical linear distribution of nuclei. In most cases, the fibromuscular stroma was less prominent and focal eosinophilic cells were observed. The remaining cases were histologically similar to the index case, with a more prominent stromal component and non-apically oriented nuclei. Thick strands of the fibromuscular bundles were also consistently noted (Supplementary File S1). Notably, the diagnostic variability in distinguishing CCPRCT from RCCFMS was considerable. The median age at diagnosis was 61 years, with a male-to-female ratio of 2:1. The available data suggest a clinical course with low malignant potential. One case was reported deceased at one year with a T1aNXM0 diagnosis, while follow-up data were unavailable for four cases. Seven of the 10 similar cases had multifocal tumors, according to the pathology report at the time of surgery. Of the 10 similar cases, eight were from patients of African American ethnicity.

In addition to their transcriptomic similarity, the identified cases also shared several clinical characteristics. Most tumors were at a low stage at diagnosis (predominantly T1), multifocality was common, metastatic disease was not reported, and the available follow-up suggested an apparently indolent clinical course. These shared clinical features supported the subsequent histopathological re-evaluation of the transcriptomically identified cases.

### 3.6. Identification and Evaluation of Potential Markers Through Transcriptomic Analysis

**Figure 5:**
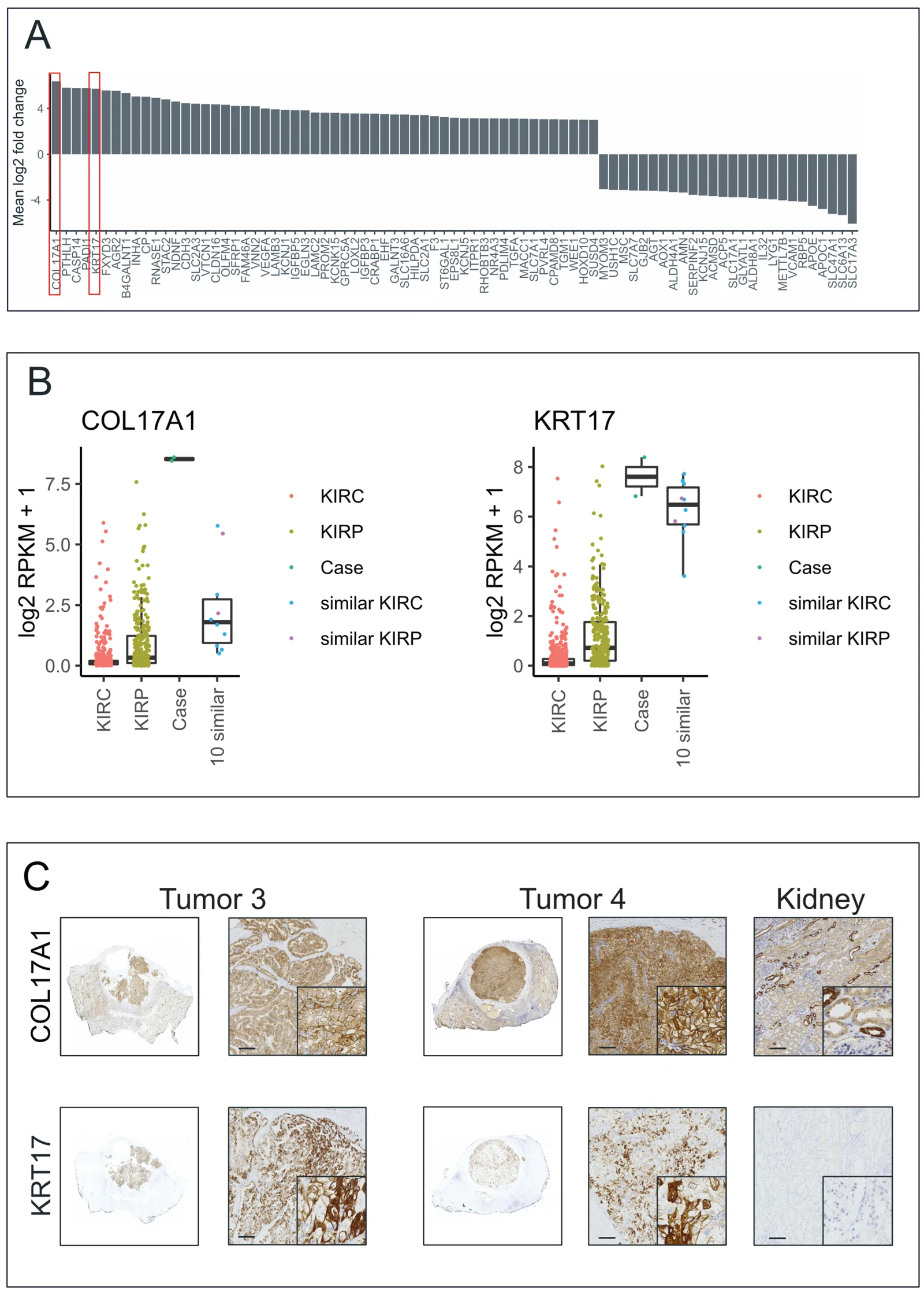
Transcriptional analysis of the index case and sub-cluster cases reveals potential markers. (**A**) Expression analysis of the identified sub-cluster including the index case identifies *COL17A1* and *KRT17* as possible markers (red box). (**B**) Transcriptional analysis of markers with significantly higher expression compared with clear cell (KIRC) and papillary RCC (KIRP). (**C**) Immunohistochemistry for COL17A1 demonstrates diffuse high expression in tumors, and high expression only in normal kidney collecting ducts. KRT17/CK17 shows diffuse high expression in tumors, and absent staining in normal kidney tissue. Scale bar = 200 μm. Magnified square = 200 × 200 μm. COL17A1, Collagen Type XVII Alpha 1 Chain; KRT17, Cytokeratin 17; RPKM, Reads per kilobase per million.

Potential markers were identified through differential analysis across genes with high and low expression. Two proteins—COL17A1 and CK17—were found to be possible diagnostic markers based on their distinctly high expression, representing structural proteins that may be suitable as targets for diagnostic antibodies (Figure 5A,B, Supplementary Tables S2 and S3). Consistent with the RNA expression, IHC of the index case demonstrated general and distinct positive staining for COL17A1 and CK17 (Figure 5C). No expression of CK17 was observed in normal kidneys, whereas COL17A1 was found to be expressed by scattered cells in the collecting ducts. To further examine the expression of COL17A1 and CK17, TMAs from 257 CCRCC cases, 68 PRCC cases, and six cases of recently diagnosed CCPRCT were stained for these proteins (Table 3), none of which showed double positive staining for CK17 and COL17A1. One case of PRCC and four cases of CCRCC were found to be positive for COL17A1. Of these, sarcomatoid histology was observed in one case and histology comparable to RCCFMS in two cases. For CCPRCT, strong diffuse positive staining for CK17 was found in 5/6 (83%) cases, while diffuse positive staining for COL17A1 was observed in 6/6 (100%) cases (Supplementary Table S5).

## 4. Discussion

In this exploratory study, we identified a case of multifocal RCC with a clear cell tubulopapillary growth pattern with thick fibromyomatous stroma and lacking apically oriented nuclei. The case also displayed “cup-shaped” CAIX positivity and diffuse CK7 positivity. These features were most consistent with the non-established diagnosis of RCCFMS, with resemblance to CCPRCT. We subjected two tumors to DNA and RNA sequencing and compared their transcriptional and genomic profiles to 885 RCC cases in the TCGA database, with the aim of identifying similar tumors in an exploratory manner. Our analysis suggests a potential biological relationship between at least some cases of RCCFMS and CCPRCT, raising the possibility that these tumors may represent variants of clear cell tumors of low malignant potential within the same spectrum; however, this hypothesis requires further validation.

The recognition of CCPRCT in the 2016 WHO classification was significant, marking the first acknowledgment of an indolent clear cell tumor [5]. Delineation of rare tumor subtypes is not always straightforward; for instance, RAT was initially described as distinct, but was later incorporated into the CCPRCT category [5,33]. In contrast, RCCFMS has been provisionally maintained as a separate entity on histological grounds, with leiomyomatous stromal bands and absence of the nuclei forming an abluminal picket fence position, thus distinguishing it from CCPRCT [2,14]. However, stromal components have been questioned as reliable diagnostic criteria, as similar features are also seen in more common RCC types such as CCRCC and PRCC [34,35]. In a cohort of 18 RCCFMS cases, Shah et al. have reported molecular alterations involving the TSC/MTOR pathway [10]. In a comparative study by Li et al., five patients with *TSC/MTOR* and seven patients with *TCEB1/ELOC* alterations were studied, both with RCCFMS morphology. Their clinical characteristics differed substantially, with a mean age of 23 years (range 19–30) and ISUP grade 2–3 in the *TSC/MTOR* patients, indicating biological variability [36]. These observations support the previous notion that RCCFMS likely represents a heterogeneous entity with similar morphology [37]. Gupta et al. have highlighted the challenges associated with applying genetic diagnostic criteria in routine pathology. They also proposed a reversion from the designation of tumor to the previous one of indolent carcinoma, which could broaden the diagnostic criteria for CCPRCT [38]. We concur with this view, particularly in cases with more advanced age, low-grade nuclei, and small tumors.

In this study, clinical data from the identified tumors indicated low tumor stage (T1), absence of metastases, and a general course commensurate with low malignant potential. One of the 10 patients had died within 5 years; in particular, this patient was categorised as T1aNXM0 and died one year after diagnosis. There is no information on their cause of death, but given the TNM status at diagnosis, it is unlikely that the cause was disseminated RCC. The available data suggest that the aggressiveness of tumors in this sub-cluster is low or very low. All cases displayed clear cell morphology, seven cases showed areas of apically oriented nuclei that would categorise them as CCPRCT, and three had features consistent with RCCFMS. Of note, leiomyomatous strands were observed not only in RCCFMS but also in several CCPRCT cases. The finding of overlapping features supports the notion that these tumors are difficult to separate in routine diagnostics.

At the molecular level, the subgroup was characterised by diploid genomes and absence of common driver mutations; this is in contrast to sporadic CCRCC, for which 3p loss and *VHL* inactivation are hallmarks [39]. RNA-seq did not reveal any increase in mTOR activity. A clear cell histology suggests involvement of the VHL/HIF pathway; in this context, downregulation of *VHL* mRNA was consistently observed, with evidence of hypermethylation at specific CpG sites. Importantly, these findings highlight that epigenetic mechanisms may contribute to the molecular phenotype of these tumors, which could underlie tumor development in this subgroup. Previous studies on epigenetic regulation of *VHL* have specifically indicated cg13672843, which is positioned at the beginning of exon 1 [18,40]. These findings raise the possibility that epigenetic regulation of *VHL* may contribute to pseudohypoxic signalling and the clear cell phenotype, although functional studies are required to establish causality. Immunohistochemistry for VHL confirmed lower VHL expression in case tissues than in normal kidney tissue. We propose that reduced *VHL* expression—potentially associated with increased DNA methylation—may contribute to pseudohypoxic realignment and a clear cell phenotype in these tumors. In addition to reduced *VHL* expression, the identified tumors demonstrated enrichment of hypoxia-related gene signatures. Although the present study does not establish the underlying mechanism, this observation is consistent with activation of pseudohypoxic signalling—a hallmark of many clear cell renal neoplasms. Together with the preserved chromosome 3p and absence of recurrent *VHL* mutations, these findings suggest that this subgroup may achieve a clear cell phenotype through mechanisms distinct from the canonical genetic inactivation of *VHL*. These findings are consistent with the hypothesis that increased DNA methylation may contribute to reduced *VHL* expression and the clear cell phenotype. Whether reduced *VHL* expression represents a primary molecular event contributing to tumor development or a secondary consequence of other biological processes cannot be determined from the present study, and further functional studies investigating the VHL/HIF signalling pathway and related molecular mechanisms are required to clarify this relationship. The enrichment of TGF-β signalling may also be biologically relevant. TGF-β is involved in extracellular matrix remodelling, stromal activation, and epithelial–mesenchymal interactions, processes that may contribute to the prominent fibromyomatous stroma observed in several tumors within the subgroup. Although this association remains speculative, it is consistent with the overlapping morphological features shared by CCPRCT and RCCFMS and provides a potential biological link between the transcriptomic findings and tumor histology.

Positivity for CK7 and diffuse “cup-shaped” CAIX positivity are shared features for RCCFMS and CCPRCT, in contrast to the “box-shaped” expression associated with CCRCC. In normal CAIX-expressing tissues such as the gall bladder epithelium, intestinal crypts, and efferent ducts of the testis, CAIX is predominantly localised to the basolateral membranes. Cup-shaped expression is therefore likely associated with normal intracellular sorting, a feature that is preserved in CCPRCT and RCCFMS. Thus, CAIX and CK7 expression analyses may be unsuitable for the differential diagnosis of CCPRCT vs. RCCFMS. E-cadherin was also expressed basolaterally in the index case, which further strengthens the assumption of a conserved cell sorting apparatus.

The index case displayed strong diffuse CD10 positivity, as well as a lack of apical alignment of nuclei. CD10 positivity has been noted, to some extent (2–24%), in previous reports of CCPRCT [41,42,43]. Two of the 10 similar tumors (TCGA-DV-5567 and TCGA-BP-4760) have also previously been highlighted as probable CCPRCT [44].

An unusual germline mutation in *TSC2* (p.Val225Gly) was identified in the index case, which was predicted to result in loss of function for the protein. In addition, sub-clonal variation appears to be present, with T3 having a nonsense mutation (stop-gain) at L619 and T4 having a splice site mutation (rs45454192). Considering the multifocality, bilateral tumor localisation, and the presence of *TSC2* alterations, a hereditary predisposition may be present in the index patient. Although this cannot be established from the current data, the possibility of an underlying hereditary risk factor should be considered, with tumor development possibly caused by a “second hit” in different ways in the two tumors examined. The *TSC2* alterations identified in the index case were not observed among the remaining transcriptomically similar tumors, and are therefore unlikely to represent defining molecular features of the subgroup identified in this study. Instead, these findings most likely reflect biological characteristics specific to the index patient. Bilateral tumors are strongly associated with hereditary kidney cancer, without necessarily belonging to any of the known hereditary syndromes [45,46]. A review of the pathology reports for each similar case showed that 7 out of 10 individuals underwent excision of multiple tumors during surgery. The clinical data also revealed a significant occurrence of other malignancies, albeit without specific details. In CCPRCT, a high incidence of bilateral disease (up to 25%) has been reported [41,47].

In the obtained cohort, we observed a high prevalence of African American ethnicity compared to the entire TCGA RCC dataset (72% vs. 13%). Interestingly, several previous studies have reported a substantial increase in the occurrence of CCPRCT among African Americans, further supporting elements of a hereditary component [41,42,48]. A similar distribution of sex and age at diagnosis was observed in the identified cases, and previous malignancies were registered in five (50%) of the 10 cases. This is an interesting observation; however, whether an increased risk of other malignancies exists in this group cannot be answered in this study.

We raise the possibility that CCPRCT and at least a subgroup of tumors with features of RCCFMS may represent morphological variants within a similar spectrum. The 2022 WHO classification reflects the increasing importance of integrating molecular features into the classification of renal tumors. Our findings support this concept by demonstrating that tumors with overlapping morphology may also share transcriptomic characteristics despite receiving different conventional diagnoses. The overlap with *ELOC*-mutated RCC further highlights the molecular heterogeneity of tumors with fibromyomatous stroma. Although exploratory, our results suggest that molecular profiling may complement conventional histopathology in diagnostically challenging cases. Positive markers for this category would be valuable; therefore, we examined the list of differentially expressed genes and the highly expressed structural proteins COL17A1 and CK17 were selected for further analysis. Unlike most other collagens, COL17A1 is a transmembrane protein. Furthermore, it is part of the multiprotein complex formed by hemidesmosomes that anchor the cell to the basal cell membrane; for instance, basal stem cells of the epidermis are anchored to the basal membrane by COL17A1 [49]. The index case was positive for CK17 and COL17A1. Although the present study focused on highly expressed candidate diagnostic markers, several genes were also found to be downregulated within the identified tumor cluster. The biological significance of these findings remains to be determined, and may provide additional insights into the molecular biology of these tumors in future studies. We also stained TMA samples from 257 CCRCC and 68 PRCC tumors, all of which (except for one case of PRCC) showed negative staining. An independent cohort of six cases of CCPRCT were also stained for CK17 and COL17A1, and all displayed positivity throughout most of the tumor tissue.

Three urological pathologists from independent universities re-evaluated the 10 similar cases based on available H&E histology. Considerable interobserver variability was noted, highlighting the challenges associated with primary diagnosis—especially regarding the distinction between RCCFMS, CCRCC, and CCPRCT. The substantial interobserver variability observed highlights the challenges associated with classifying these uncommon clear cell renal tumors from their morphology alone. Although characteristic features such as apically oriented nuclei, cup-shaped CAIX staining, and the extent of fibromyomatous stroma are incorporated into current diagnostic criteria, these features may overlap and are subject to interpretation, particularly in borderline cases. The observed variability between the experienced uropathologists suggests that morphology alone may not always provide sufficient diagnostic certainty. Our findings support the concept that molecular profiling may provide complementary information in diagnostically challenging cases. Rather than replacing conventional histopathology, transcriptomic and genomic analysis may help in identifying biologically similar tumors that transcend traditional morphological categories and thereby improve diagnostic confidence. The identified markers may possibly aid in differentiating CCRCC cases from cases with low malignant potential, which could be of particular importance when diagnosing biopsy material with limited tumor content.

The strengths of this study include performing DNA- and RNA-seq to a very high depth, which is a key factor when applying comparative sequencing-based analyses [50]. Although the cluster formation approach was unsupervised and relied on transcriptional similarity to the included case, the identified tumors were found to share many morphological and clinical traits, further supporting their resemblance. Although the index case and TCGA RNA-seq data were processed using the same bioinformatic workflow, differences in sequencing platforms, library preparation, and sample processing may introduce batch effects that cannot be completely excluded. Consequently, the transcriptomic clustering should be interpreted as exploratory and requires validation in independent cohorts. Patients from several different centres with different platforms over several years are included in TCGA, and this potential source of analytical error has previously been pointed out [15,51]. Although the study was initiated from a single index case, the principal objective was to use this case as a discovery tool to identify transcriptomically related tumors within a large independent dataset. Consequently, the biological observations were derived from a combined analysis of this molecularly defined cohort rather than from the index case alone.

This study has several important limitations. First, the analysis was centred on a single index case, and the identification of similar tumors in the TCGA dataset was based on an exploratory, transcriptome-driven approach, which may be sensitive to methodological and batch-related variation. Second, the lack of histological material for TCGA tumors precludes confirmatory immunohistochemistry. Third, the immunohistochemical validation of CK17 and COL17A1 was limited by the small number of independent CCPRCT cases and the absence of several rare renal tumor entities that may enter the differential diagnosis. Although the markers showed high expression in the index case and in the small CCPRCT cohort, and were largely absent from the clear cell and papillary RCC tissue micro-arrays, these findings do not establish diagnostic specificity across the full spectrum of renal neoplasia. In particular, further evaluation is required in *ELOC*-mutated RCC, *TSC1/TSC2/MTOR*-altered RCC with fibromyomatous stroma, chromophobe RCC, SDH-deficient RCC, FH-deficient RCC, and eosinophilic solid and cystic RCC. Therefore, CK17 and COL17A1 should be regarded as candidate markers pending validation in larger, independently characterised cohorts. Fourth, clinical follow-up data for several TCGA cases were incomplete, restricting the assessment of biological behaviours. Furthermore, although increased *VHL* methylation was associated with reduced gene expression, the present study does not establish a causal relationship and functional studies will be required to determine whether methylation directly regulates *VHL* expression or contributes to tumor development. Taken together, these limitations indicate that the findings should be interpreted as hypothesis-generating and require validation in larger, prospectively characterised cohorts.

## 5. Conclusions

We identified a cluster of indolent renal tumors with clear cell morphology from the TCGA database by comparing whole-genome sequencing data from an index case with overlapping features of CCPRCT and the non-established subtype RCCFMS. The TCGA cluster showed similar clinical characteristics, with morphology closely resembling CCPRCT and RCCFMS. The tumors lacked identifiable driver mutations and exhibited downregulation of *VHL*, associated with increased methylation of selected *VHL* CpG sites. They also demonstrated enrichment of hypoxia and TGF-β-related transcriptional programmes, together supporting a distinct molecular phenotype. Our findings suggest that CCPRCT could share important molecular and morphological traits with a subset of tumors best described as RCCFMS without major genetic alterations. Our results should be regarded as hypothesis-generating and exploratory, warranting further studies in larger cohorts for validation. Importantly, CK17 and COL17A1 emerged as candidate diagnostic markers that may aid in the recognition of tumors in this subgroup. The notable interobserver variability in the histological re-evaluation of the TCGA cases further highlights the need for complementary molecular approaches when evaluating diagnostically challenging clear cell renal tumors.

## Figures and Tables

**Table 1:** Clinical overview of index case and the 10 similar cases.

Patient	Sex/Age	TNM Stage	Multiple Tumors (nr)	Prior Malignancy	Year of Diagnosis	Ethnicity	5-Year OS
Index Case	M/50	T1a N0M0	Yes (6)	No	2016	CC	Yes
1	M/61	T1a NXM0	No	Yes	2012	AA	LTFU
2	M/63	T1a NXM0	Yes (2)	No	2005	CC	Yes
3	M/69	T1a NXM0	Yes (3)	Yes	2004	AA	Yes
4	F/40	T1a N0M0	Yes (11)	Yes	2008	AA	Yes
5	M/62	T1 NXM0 ^a^	No	No	2010	CC	LTFU
6	M/53	T1a NXMX	Yes (3)	No	2007	AA	LTFU
7	M/55	T1b NXM0	Yes (9)	No	2008	AA	Yes
8	F/55	T1a NXM0	Yes (3)	Yes	2008	AA	No
9	M/77	T1a N0M0	Yes (2)	Yes	2006	AA	Yes
10	F/74	T1a N0M0	No	No	2011	AA	LTFU

M, male; F, female; TCGA, The Cancer Genome Atlas; CC, Caucasian; AA, African American; OS, overall survival; LTFU, lost to follow-up. ^a^ T1a or b not specified.

**Table 2:** Primary histology and re-evaluation of the 10 similar cases.

Patient	Sex/Age	Primary Classification in TCGA	Flagged in Previous Marker Studies ^a^	Histology Re-Evaluation (M.J.)	Histology Re-Evaluation (M.L.)	Histology Re-Evaluation (N.M.)
Index Case	M/50	-	-	RCCFMS	RCCFMS	RCCFMS
1	M/61	RCC NOS	No	CCPRCT	CCPRCT	RCCFMS
2	M/63	CCRCC	Yes ^1^	RCCFMS	CCRCC	CCRCC
3	M/69	CCRCC	Yes ^2^	CCPRCT	CCPRCT	RCCFMS
4	F/40	CCRCC	No	RCCFMS	CCRCC	RCCFMS
5	M/62	CCRCC	Yes ^3^	CCPRCT	CCRCC	RCCFMS
6	M/53	CCRCC	No	RCCFMS	RCCFMS	RCCFMS
7	M/55	CCRCC	No	CCPRCT	RCCFMS	RCCFMS
8	F/55	CCRCC	No	CCPRCT	RCCFMS	RCC NOS
9	M/77	PRCC	No	CCPRCT	CCPRCT	PRCC
10	F/74	PRCC	Yes ^4^	RCCFMS	CCRCC	PRCC

M, male; F, female; TCGA, The Cancer Genome Atlas; RCC, renal cell carcinoma; NOS, not otherwise specified; CCRCC, clear cell RCC; PRCC, papillary RCC; M.J., Martin E. Johansson; M.L., Martin Lindgren; N.M., Niels Marcussen. ^a^ Chen et al. Multilevel Genomics-Based Taxonomy of Renal Cell Carcinoma [18]. ^1^ “Not Clear Cell or Chromophobe on histopathological re-review”. ^2^ “Not Clear Cell or Chromophobe on histopathological re-review”. ^3^ “Suspect molecular profile”. ^4^ “Not Papillary-Possibly CCRCC”.

**Table 3:** Positive staining pattern for CK17 and COL17A1.

	No. of Cases	CK17 (%)	COL17A1 (%)
TMA CCRCC	257	1 (<1) ^a^	4 (1.6) ^b^
TMA PRCC	68	1 (1.5)	1 (1.5) ^c^
CCPRCT	6	5 (83)	6 (100)

TMA, tissue microarray; CCRCC, clear cell RCC; PRCC, papillary RCC; CCPRCT, clear cell papillary renal cell tumor; CK17, cytokeratin 17; COL17A1, Collagen Type XVII Alpha 1 Chain. ^a^ Sarcomatoid clear cell RCC. ^b^ One case with sarcomatoid features. One case compatible with RCC with leiomyomatous stroma. ^c^ Histology compatible with RCC with leiomyomatous stroma.

## Data Availability

The original contributions presented in this study are included in the article/Supplementary Materials. Further inquiries can be directed to the corresponding author.

## Supplementary Materials

The following supporting information can be downloaded at: https://www.mdpi.com/article/10.3390/cancers18162713/s1, Figure S1: Gene set enrichment analysis of T3 and T4; Figure S2: Schematic flowchart of the identification and evaluation of TCGA tumors. WGS, whole-genome sequencing; TCGA, The Cancer Genome Atlas Program; t-SNE, t-distributed stochastic neighbor embedding. Figure S3: Immunohistochemistry of Tumor 3, Tumor 4, and normal kidney cortex. Scale bar = 200 μm. Magnified square = 200 × 200 μm. TSC2, Tuberous Sclerosis Complex 2; p-mTOR, phospho-Mechanistic Target Of Rapamycin Kinase; pS6K1, phospho-S6 Kinase 1. Table S1: IHC results and antibodies used. Ready to use (RTU). Table S2: RNA expression values of KIRC vs. our case. Column H is displayed in Figure 5A. Table S3: RNA expression values of KIRP vs. our case. Gene set enrichment of T3 and T4. Table S4: The unique identification in TCGA of the 10 similar patients to our case. Table S5: Staining pattern of five cases of clear cell papillary renal cell tumors (CCPRCT). Table S6: The Cancer Genome Atlas—Abbreviations. File S1: Histological slides from the 10 identified similar cases.

## Abbreviations

The following abbreviations are used in this manuscript:

AE1-3	Epithelial antibody cocktail 1-3
BAM	Binary Alignment Map
CAIX	Carbonic anhydrase IX
CCPRCT	Clear cell papillary renal cell tumor
CCRCC	Clear cell renal cell carcinoma
CD10	Cluster of differentiation 10
CHRCC	Chromophobe renal cell carcinoma
COL17A1	Collagen Type XVII Alpha 1 Chain
COSMIC	Catalogue Of Somatic Mutations In Cancer
CpG	Cytosine–phosphate–Guanine
dbSNP	Single Nucleotide Polymorphism Database
Dnr	Registry number
ECAD	E-cadherin
ELOC	Elongin C
EMA	Epithelial membrane antigen
ESP	Exome Sequencing Project
GATK	Genome Analysis Toolkit
GRCh37	Genome Reference Consortium Human Build 37
GSVA	Gene Set Variation Analysis
H&E	Haematoxylin and eosin
IHC	Immunohistochemistry
KRT17/CK17	Cytokeratin 17
KRT7/CK7	Cytokeratin 7
ML	Martin Lindström
MJ	Martin E. Johansson
MSigDB	Molecular Signatures Database
MTOR	Mammalian target of rapamycin
p-mTOR	Phospho-Mechanistic Target of Rapamycin Kinase
p-S6K1	Phospho-S6 Kinase 1
PRCC	Papillary renal cell carcinoma
RAT	Renal angiomyomatous tumor
RCC	Renal cell carcinoma
RCCFMS	Renal cell carcinoma with prominent fibromyomatous stroma
RJ	Rasmus Jakobsson
RNA	Ribonucleic acid
RPKM	Reads per kilobase per million
SAM	Sequence Alignment Map
TCGA	The Cancer Genome Atlas
TGF-β	Transforming growth factor beta
TMA	Tissue microarray
TSC1	Tuberous Sclerosis Complex 1
TSC2	Tuberous Sclerosis Complex 2
t-SNE	t-distributed stochastic neighbour embedding
UCSC	University of California Santa Cruz
VEP	Variant Effect Predictor
VHL	von Hippel–Lindau tumor suppressor
WGS	Whole-genome sequencing
WHO	The World Health Organisation

## References

[1] Kovacs G., Akhtar M., Beckwith B.J., Bugert P., Cooper C.S., Delahunt B., Eble J.N., Fleming S., Ljungberg B., Medeiros L.J. (1997). The Heidelberg classification of renal cell tumors. J. Pathol..

[2] Moch H., Amin M.B., Berney D.M., Comperat E.M., Gill A.J., Hartmann A., Menon S., Raspollini M.R., Rubin M.A., Srigley J.R. (2022). The 2022 World Health Organization Classification of Tumors of the Urinary System and Male Genital Organs-Part A: Renal, Penile, and Testicular Tumors. Eur. Urol..

[3] Trpkov K., Hes O., Williamson S.R., Adeniran A.J., Agaimy A., Alaghehbandan R., Amin M.B., Argani P., Chen Y.B., Cheng L. (2021). New developments in existing WHO entities and evolving molecular concepts: The Genitourinary Pathology Society (GUPS) update on renal neoplasia. Mod. Pathol..

[4] Eble J.N. (2021). Contributions of genetics to the evolution of the diagnostic classification of renal cell neoplasia: A personal perspective. Pathology.

[5] Moch H., Cubilla A.L., Humphrey P.A., Reuter V.E., Ulbright T.M. (2016). The 2016 WHO Classification of Tumors of the Urinary System and Male Genital Organs-Part A: Renal, Penile, and Testicular Tumors. Eur. Urol..

[6] Michal M., Hes O., Havlicek F. (2000). Benign renal angiomyoadenomatous tumor: A previously unreported renal tumor. Ann. Diagn. Pathol..

[7] Adam J., Couturier J., Molinie V., Vieillefond A., Sibony M. (2011). Clear-cell papillary renal cell carcinoma: 24 cases of a distinct low-grade renal tumor and a comparative genomic hybridization array study of seven cases. Histopathology.

[8] Bhatnagar R., Alexiev B.A. (2012). Renal-cell carcinomas in end-stage kidneys: A clinicopathological study with emphasis on clear-cell papillary renal-cell carcinoma and acquired cystic kidney disease-associated carcinoma. Int. J. Surg. Pathol..

[9] Shah R.B. (2022). Renal Cell Carcinoma with Fibromyomatous Stroma-The Whole Story. Adv. Anat. Pathol..

[10] Shah R.B., Stohr B.A., Tu Z.J., Gao Y., Przybycin C.G., Nguyen J., Cox R.M., Rashid-Kolvear F., Weindel M.D., Farkas D.H. (2020). “Renal Cell Carcinoma with Leiomyomatous Stroma” Harbor Somatic Mutations of TSC1, TSC2, MTOR, and/or ELOC (TCEB1): Clinicopathologic and Molecular Characterization of 18 Sporadic Tumors Supports a Distinct Entity. Am. J. Surg. Pathol..

[11] Gupta S., McCarthy M.R., Tjota M.Y., Antic T., Cheville J.C. (2024). Metastatic renal cell carcinoma with fibromyomatous stroma associated with tuberous sclerosis or MTOR, TSC1/TSC2-Mutations: A Series of 4 cases and a review of the literature. Hum. Pathol..

[12] Zhu Y., Fang M., Wang S. (2025). ELOC(TCEB1)-mutated renal cell carcinoma: A case report and clinicopathological analysis. Front. Oncol..

[13] Williamson S.R., Gupta N.S., Eble J.N., Rogers C.G., Michalowski S., Zhang S., Wang M., Grignon D.J., Cheng L. (2015). Clear Cell Renal Cell Carcinoma with Borderline Features of Clear Cell Papillary Renal Cell Carcinoma: Combined Morphologic, Immunohistochemical, and Cytogenetic Analysis. Am. J. Surg. Pathol..

[14] Trpkov K., Williamson S.R., Gill A.J., Adeniran A.J., Agaimy A., Alaghehbandan R., Amin M.B., Argani P., Chen Y.B., Cheng L. (2021). Novel, emerging and provisional renal entities: The Genitourinary Pathology Society (GUPS) update on renal neoplasia. Mod. Pathol..

[15] The Cancer Genome Atlas Research Network (2013). Comprehensive molecular characterization of clear cell renal cell carcinoma. Nature.

[16] (2016). The Cancer Genome Atlas Research Network. Comprehensive Molecular Characterization of Papillary Renal-Cell Carcinoma. N. Engl. J. Med..

[17] Davis C.F., Ricketts C.J., Wang M., Yang L., Cherniack A.D., Shen H., Buhay C., Kang H., Kim S.C., Fahey C.C. (2014). The somatic genomic landscape of chromophobe renal cell carcinoma. Cancer Cell.

[18] Chen F., Zhang Y., Senbabaoglu Y., Ciriello G., Yang L., Reznik E., Shuch B., Micevic G., De Velasco G., Shinbrot E. (2016). Multilevel Genomics-Based Taxonomy of Renal Cell Carcinoma. Cell Rep..

[19] Kim D., Langmead B., Salzberg S.L. (2015). HISAT: A fast spliced aligner with low memory requirements. Nat. Methods.

[20] Anders S., Pyl P.T., Huber W. (2015). HTSeq—A Python framework to work with high-throughput sequencing data. Bioinformatics.

[21] Bagge R.O., Demir A., Karlsson J., Alaei-Mahabadi B., Einarsdottir B.O., Jespersen H., Lindberg M.F., Muth A., Nilsson L.M., Persson M. (2018). Mutational Signature and Transcriptomic Classification Analyses as the Decisive Diagnostic Tools for a Cancer of Unknown Primary. JCO Precis. Oncol..

[22] Van Der Maaten L., Hinton G. (2008). Visualizing data using t-SNE. J. Mach. Learn. Res..

[23] Liberzon A., Birger C., Thorvaldsdottir H., Ghandi M., Mesirov J.P., Tamayo P. (2015). The Molecular Signatures Database (MSigDB) hallmark gene set collection. Cell Syst..

[24] Korotkevich G., Sukhov V., Budin N., Shpak B., Artyomov M.N., Sergushichev A. (2021). Fast gene set enrichment analysis. bioRxiv.

[25] Li H., Durbin R. (2009). Fast and accurate short read alignment with Burrows-Wheeler transform. Bioinformatics.

[26] McKenna A., Hanna M., Banks E., Sivachenko A., Cibulskis K., Kernytsky A., Garimella K., Altshuler D., Gabriel S., Daly M. (2010). The Genome Analysis Toolkit: A MapReduce framework for analyzing next-generation DNA sequencing data. Genome Res..

[27] Benjamin D., Sato T., Cibulskis K., Getz G., Stewart C., Lichtenstein L. (2019). Calling Somatic SNVs and Indels with Mutect2. bioRxiv.

[28] Karczewski K.J., Francioli L.C., Tiao G., Cummings B.B., Alföldi J., Wang Q., Collins R.L., Laricchia K.M., Ganna A., Birnbaum D.P. (2020). The mutational constraint spectrum quantified from variation in 141,456 humans. Nature.

[29] Adzhubei I.A., Schmidt S., Peshkin L., Ramensky V.E., Gerasimova A., Bork P., Kondrashov A.S., Sunyaev S.R. (2010). A method and server for predicting damaging missense mutations. Nat. Methods.

[30] Sim N.L., Kumar P., Hu J., Henikoff S., Schneider G., Ng P.C. (2012). SIFT web server: Predicting effects of amino acid substitutions on proteins. Nucleic Acids Res..

[31] Ricketts C.J., De Cubas A.A., Fan H., Smith C.C., Lang M., Reznik E., Bowlby R., Gibb E.A., Akbani R., Beroukhim R. (2018). The Cancer Genome Atlas Comprehensive Molecular Characterization of Renal Cell Carcinoma. Cell Rep..

[32] Gutman D.A., Khalilia M., Lee S., Nalisnik M., Mullen Z., Beezley J., Chittajallu D.R., Manthey D., Cooper L.A.D. (2017). The Digital Slide Archive: A Software Platform for Management, Integration, and Analysis of Histology for Cancer Research. Cancer Res..

[33] Michal M., Hes O., Nemcova J., Sima R., Kuroda N., Bulimbasic S., Franco M., Sakaida N., Danis D., Kazakov D.V. (2009). Renal angiomyoadenomatous tumor: Morphologic, immunohistochemical, and molecular genetic study of a distinct entity. Virchows Arch..

[34] Petersson F., Branzovsky J., Martinek P., Korabecna M., Kruslin B., Hora M., Peckova K., Bauleth K., Pivovarcikova K., Michal M. (2014). The leiomyomatous stroma in renal cell carcinomas is polyclonal and not part of the neoplastic process. Virchows Arch..

[35] Hes O., Comperat E.M., Rioux-Leclercq N. (2016). Clear cell papillary renal cell carcinoma, renal angiomyoadenomatous tumor, and renal cell carcinoma with leiomyomatous stroma relationship of 3 types of renal tumors: A review. Ann. Diagn. Pathol..

[36] Li H., Argani P., Halper-Stromberg E., Lotan T.L., Merino M.J., Reuter V.E., Matoso A. (2023). Positive GPNMB Immunostaining Differentiates Renal Cell Carcinoma With Fibromyomatous Stroma Associated with TSC1/2/MTOR Alterations from Others. Am. J. Surg. Pathol..

[37] Trpkov K., Hes O. (2019). New and emerging renal entities: A perspective post-WHO 2016 classification. Histopathology.

[38] Gupta S., Dasari S., Sharma V., Atwell T.D., Sivasankaran G., Smoley S.A., Hardcastle J.J., Lohse C.M., Tekin B., Jimenez R.E. (2024). Metastatic clear cell papillary renal cell ‘tumor’. Histopathology.

[39] Hsieh J.J., Le V.H., Oyama T., Ricketts C.J., Ho T.H., Cheng E.H. (2018). Chromosome 3p Loss-Orchestrated VHL, HIF, and Epigenetic Deregulation in Clear Cell Renal Cell Carcinoma. J. Clin. Oncol..

[40] Zhang Y., Yang L., Kucherlapati M., Chen F., Hadjipanayis A., Pantazi A., Bristow C.A., Lee E.A., Mahadeshwar H.S., Tang J. (2018). A Pan-Cancer Compendium of Genes Deregulated by Somatic Genomic Rearrangement across More Than 1,400 Cases. Cell Rep..

[41] Weng S., DiNatale R.G., Silagy A., Mano R., Attalla K., Kashani M., Weiss K., Benfante N.E., Winer A.G., Coleman J.A. (2021). The Clinicopathologic and Molecular Landscape of Clear Cell Papillary Renal Cell Carcinoma: Implications in Diagnosis and Management. Eur. Urol..

[42] Alexiev B.A., Drachenberg C.B. (2014). Clear cell papillary renal cell carcinoma: Incidence, morphological features, immunohistochemical profile, and biologic behavior: A single institution study. Pathol. Res. Pract..

[43] Aron M., Chang E., Herrera L., Hes O., Hirsch M.S., Comperat E., Camparo P., Rao P., Picken M., Michal M. (2015). Clear cell-papillary renal cell carcinoma of the kidney not associated with end-stage renal disease: Clinicopathologic correlation with expanded immunophenotypic and molecular characterization of a large cohort with emphasis on relationship with renal angiomyoadenomatous tumor. Am. J. Surg. Pathol..

[44] Xu J., Reznik E., Lee H.J., Gundem G., Jonsson P., Sarungbam J., Bialik A., Sanchez-Vega F., Creighton C.J., Hoekstra J. (2019). Abnormal oxidative metabolism in a quiet genomic background underlies clear cell papillary renal cell carcinoma. eLife.

[45] Jakobsson R.G., Nasic S., Bratt O., Johansson M.E., Grenabo Bergdahl A. (2024). Family History and Risk of Renal Cell Carcinoma: A National Multiregister Case-Control Study. J. Urol..

[46] Ljungberg B., Albiges L., Abu-Ghanem Y., Bedke J., Capitanio U., Dabestani S., Fernandez-Pello S., Giles R.H., Hofmann F., Hora M. (2022). European Association of Urology Guidelines on Renal Cell Carcinoma: The 2022 Update. Eur. Urol..

[47] Williamson S.R. (2021). Clear cell papillary renal cell carcinoma: An update after 15 years. Pathology.

[48] Wang K., Zarzour J., Rais-Bahrami S., Gordetsky J. (2017). Clear Cell Papillary Renal Cell Carcinoma: New Clinical and Imaging Characteristics. Urology.

[49] Thangavelu P.U., Krenacs T., Dray E., Duijf P.H. (2016). In epithelial cancers, aberrant COL17A1 promoter methylation predicts its misexpression and increased invasion. Clin. Epigenet..

[50] Sims D., Sudbery I., Ilott N.E., Heger A., Ponting C.P. (2014). Sequencing depth and coverage: Key considerations in genomic analyses. Nat. Rev. Genet..

[51] Whalley J.P., Buchhalter I., Rheinbay E., Raine K.M., Stobbe M.D., Kleinheinz K., Werner J., Beltran S., Gut M., Hübschmann D. (2020). Framework for quality assessment of whole genome cancer sequences. Nat. Commun..

