# Supplementary material for: Transcriptomic Profiling Identifies a Subset of Renal Tumors with Overlapping Features of Clear Cell Papillary Renal Cell Tumor and Renal Cell Carcinoma with Fibromyomatous Stroma
Source: Cancers (Basel). 2026 Aug 21;18(16):2713. doi: 10.3390/cancers18162713 (PMC13511157; doi:10.3390/cancers18162713)
Supplement: Supplementary file 1 [file cancers-18-02713-s001.zip › Figure S2_Flowchart.pdf]

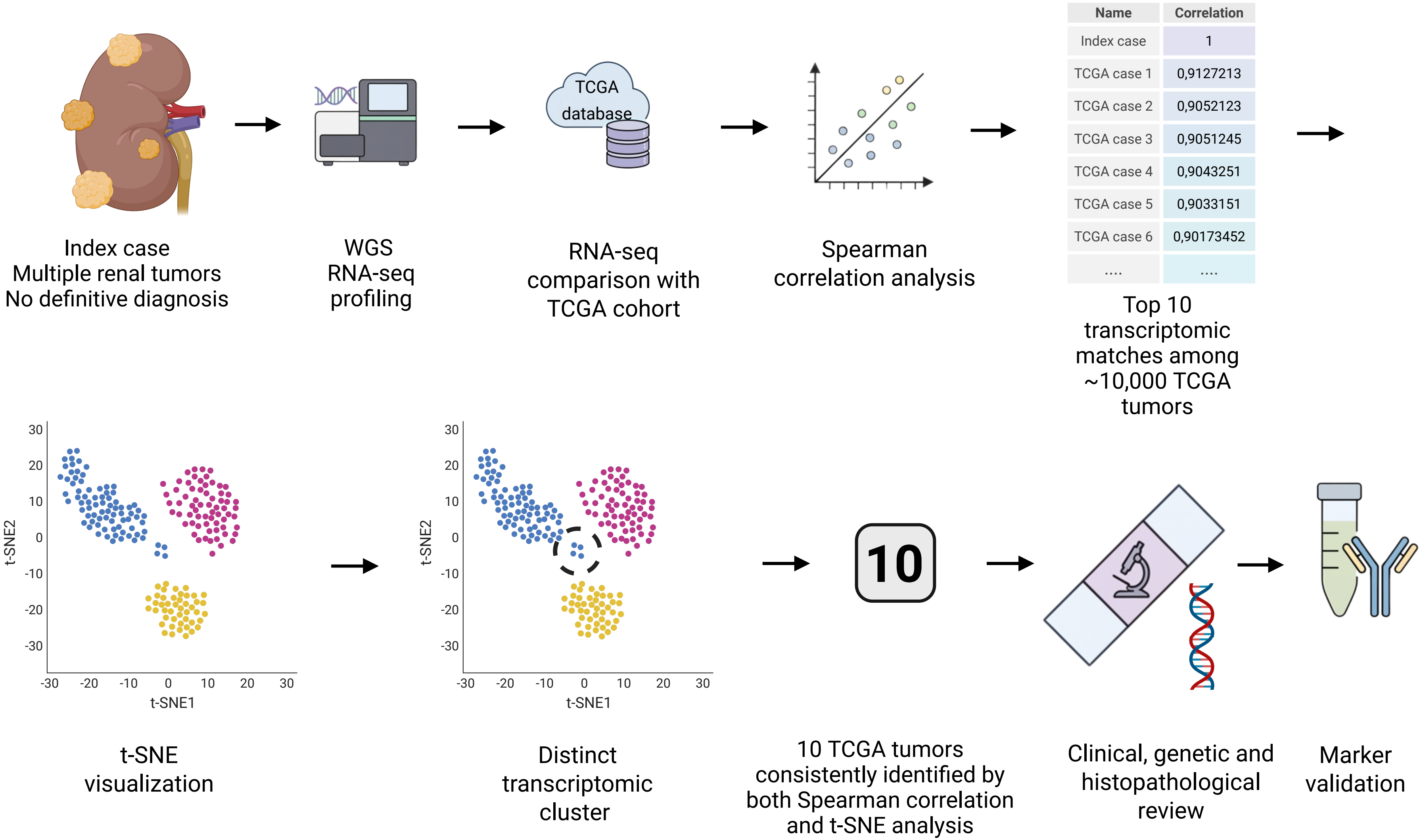

Figure S2. Schematic flowchart of the identification and evaluation of TCGA tumors. WGS, whole-genome sequencing; TCGA, The Cancer Genome Atlas Program; t-SNE, t-distributed stochastic neighbor embedding.

Created in https://BioRender.com with permission.
