# Supplementary material for: Transcriptomic Profiling Identifies a Subset of Renal Tumors with Overlapping Features of Clear Cell Papillary Renal Cell Tumor and Renal Cell Carcinoma with Fibromyomatous Stroma
Source: Cancers (Basel). 2026 Aug 21;18(16):2713. doi: 10.3390/cancers18162713 (PMC13511157; doi:10.3390/cancers18162713)
Supplement: Supplementary file 1 [file cancers-18-02713-s001.zip › Figure S3_mTOR_IHC.pdf]

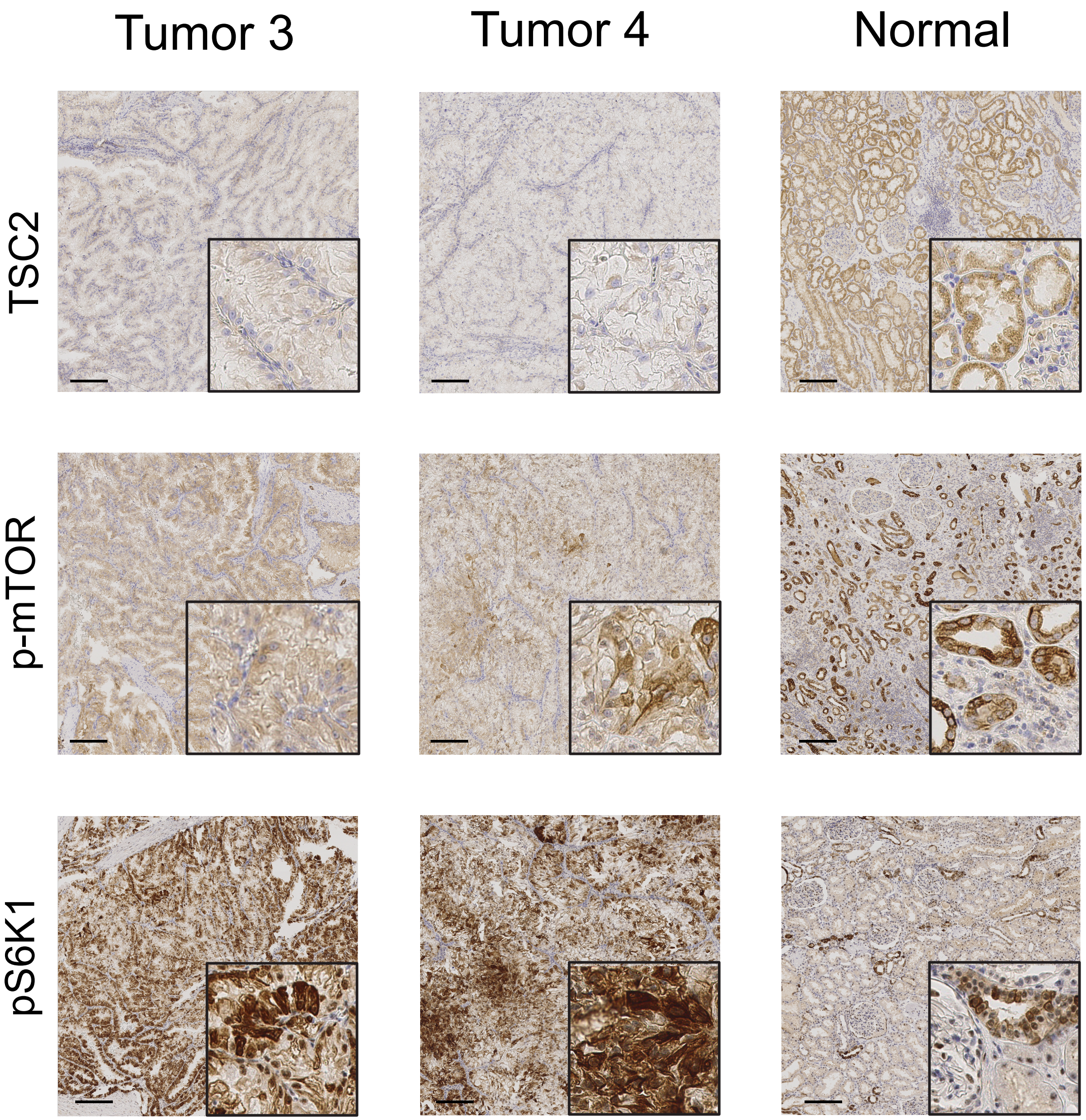

Figure S3. Immunohistochemistry of Tumor 3, Tumor 4, and normal kidney cortex. Scale bar = *200 µm*. Magnified square = *200 x 200 µm*. *TSC2,* Tuberous Sclerosis Complex 2; *p-mTOR,* phospho-Mechanistic Target Of Rapamycin Kinase; *pS6K1,* phospho-S6 Kinase 1
