# Supplementary material for: Transcriptomic Profiling Identifies a Subset of Renal Tumors with Overlapping Features of Clear Cell Papillary Renal Cell Tumor and Renal Cell Carcinoma with Fibromyomatous Stroma
Source: Cancers (Basel). 2026 Aug 21;18(16):2713. doi: 10.3390/cancers18162713 (PMC13511157; doi:10.3390/cancers18162713)
Supplement: Supplementary file 1 [file cancers-18-02713-s001.zip › File S1.pdf]

File S1. Histological slides from the 10 identified similar cases

Use TCGA database link to find full slides - Use the link and identification nr listed below; https://portal.gdc.cancer.gov/analysis_page?app=IDCViewerApp

Example slides:

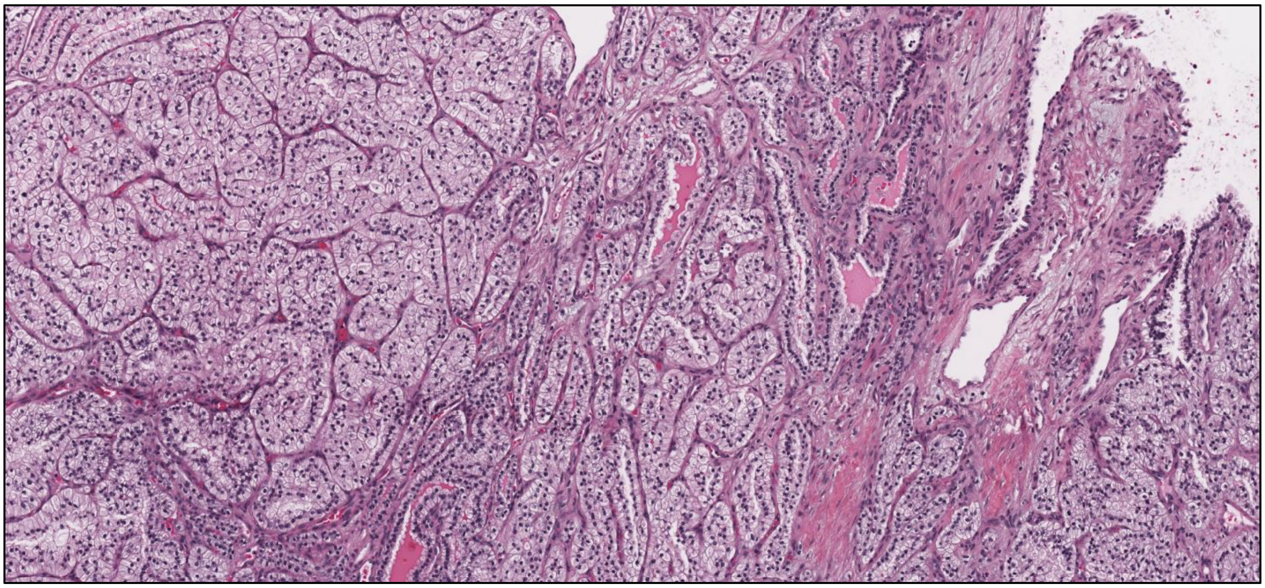

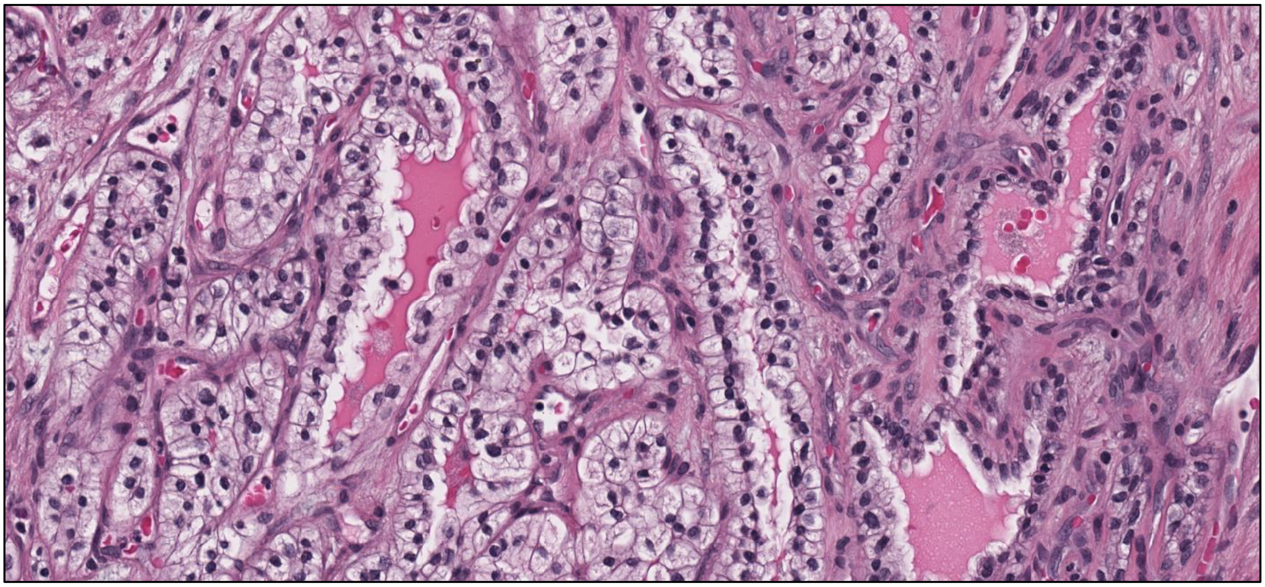

Patient 1. TCGA-B8-A54K

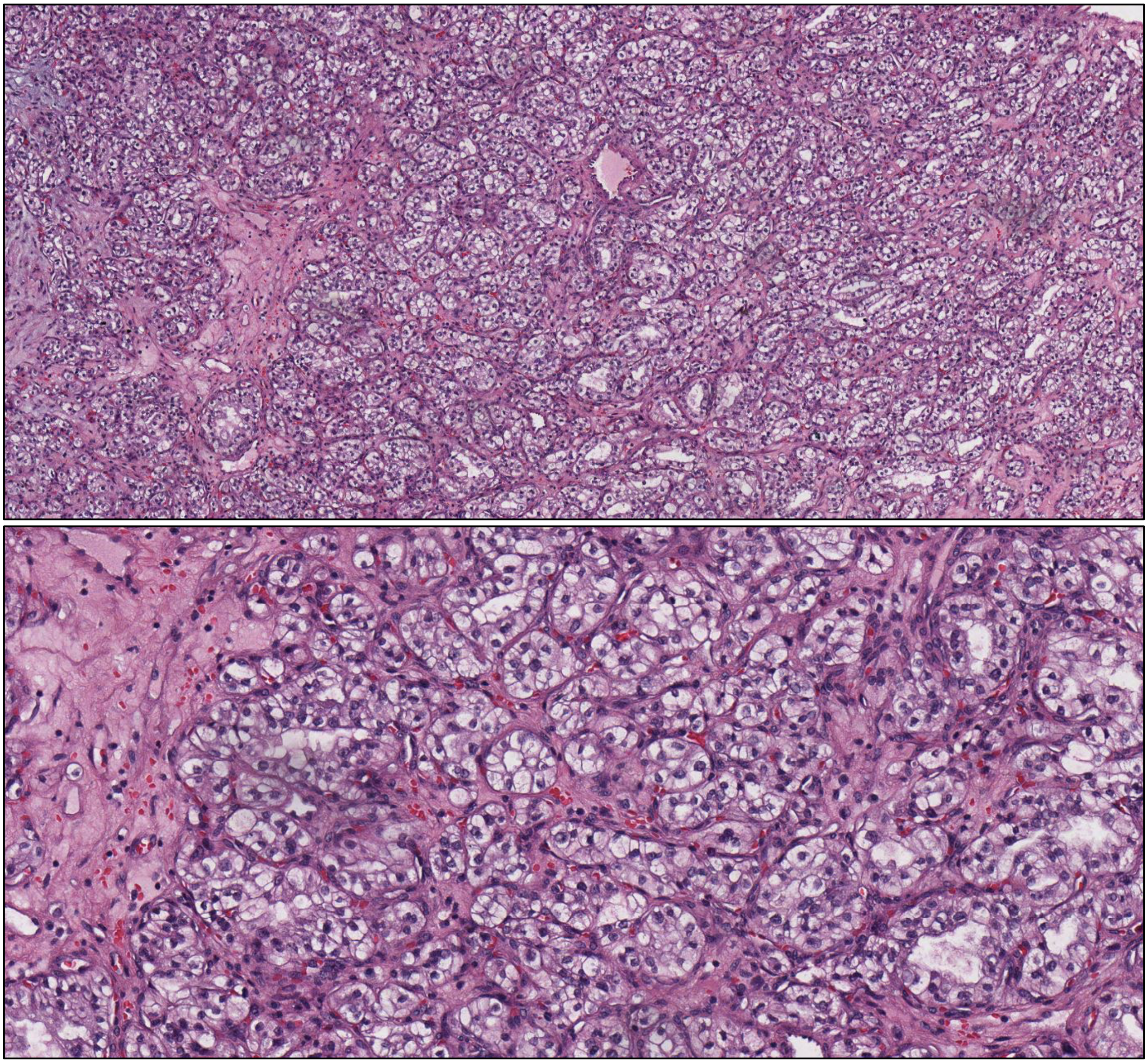

Patient 2. TCGA-BP-4769

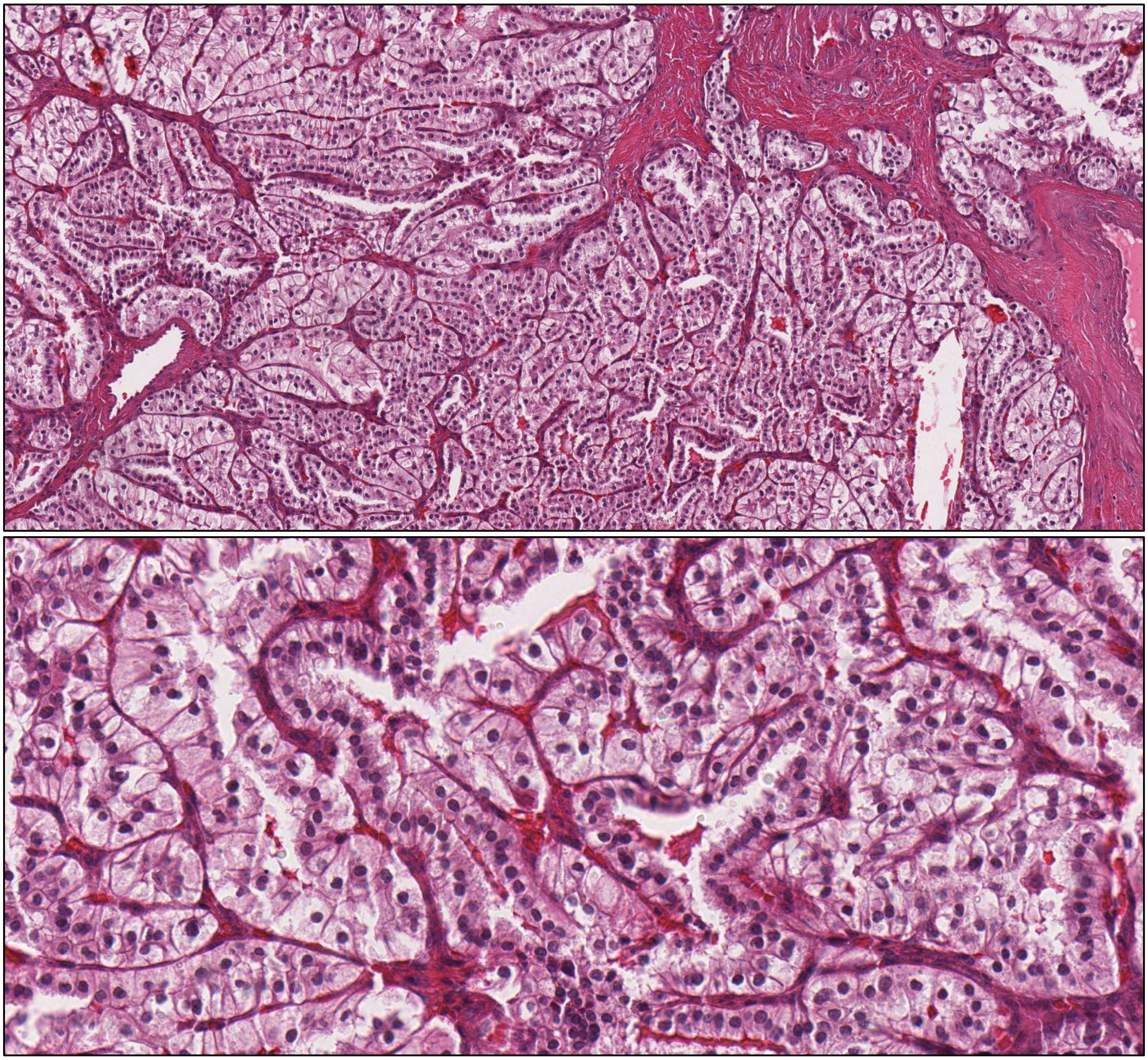

Patient 3. TCGA-BP-4760

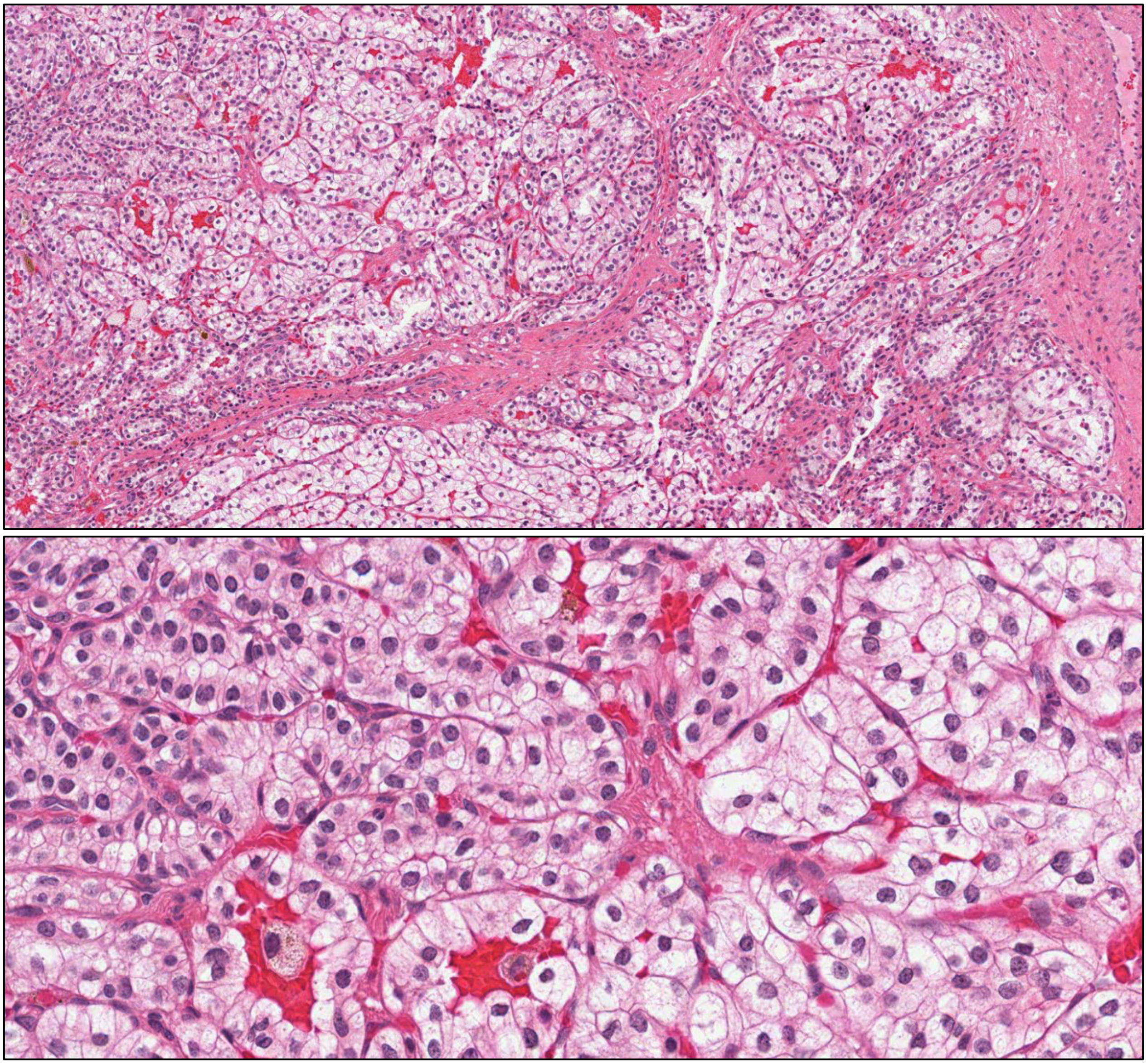

Patient 4. TCGA-DV-5567

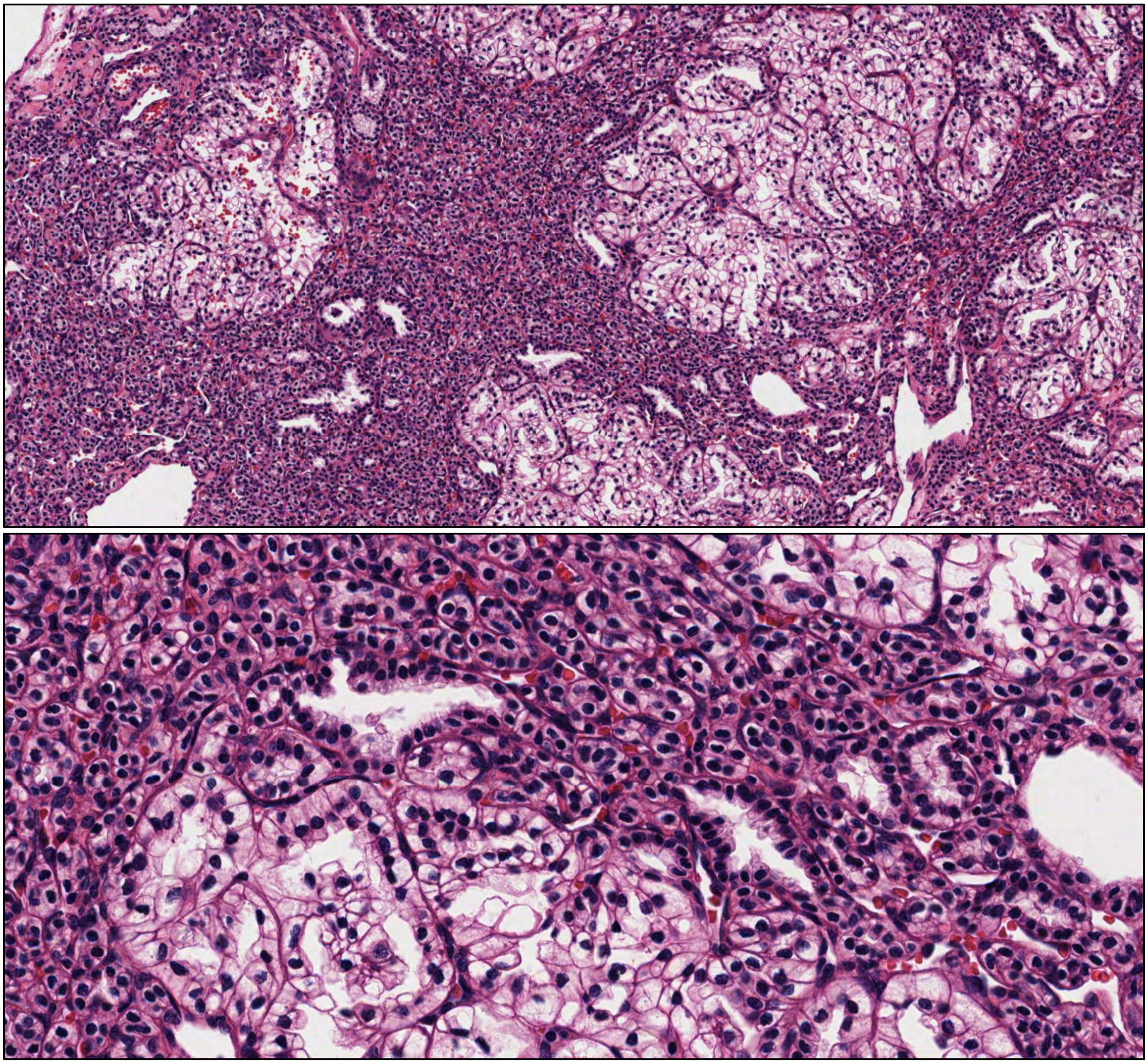

Patient 5. TCGA-B4-5378

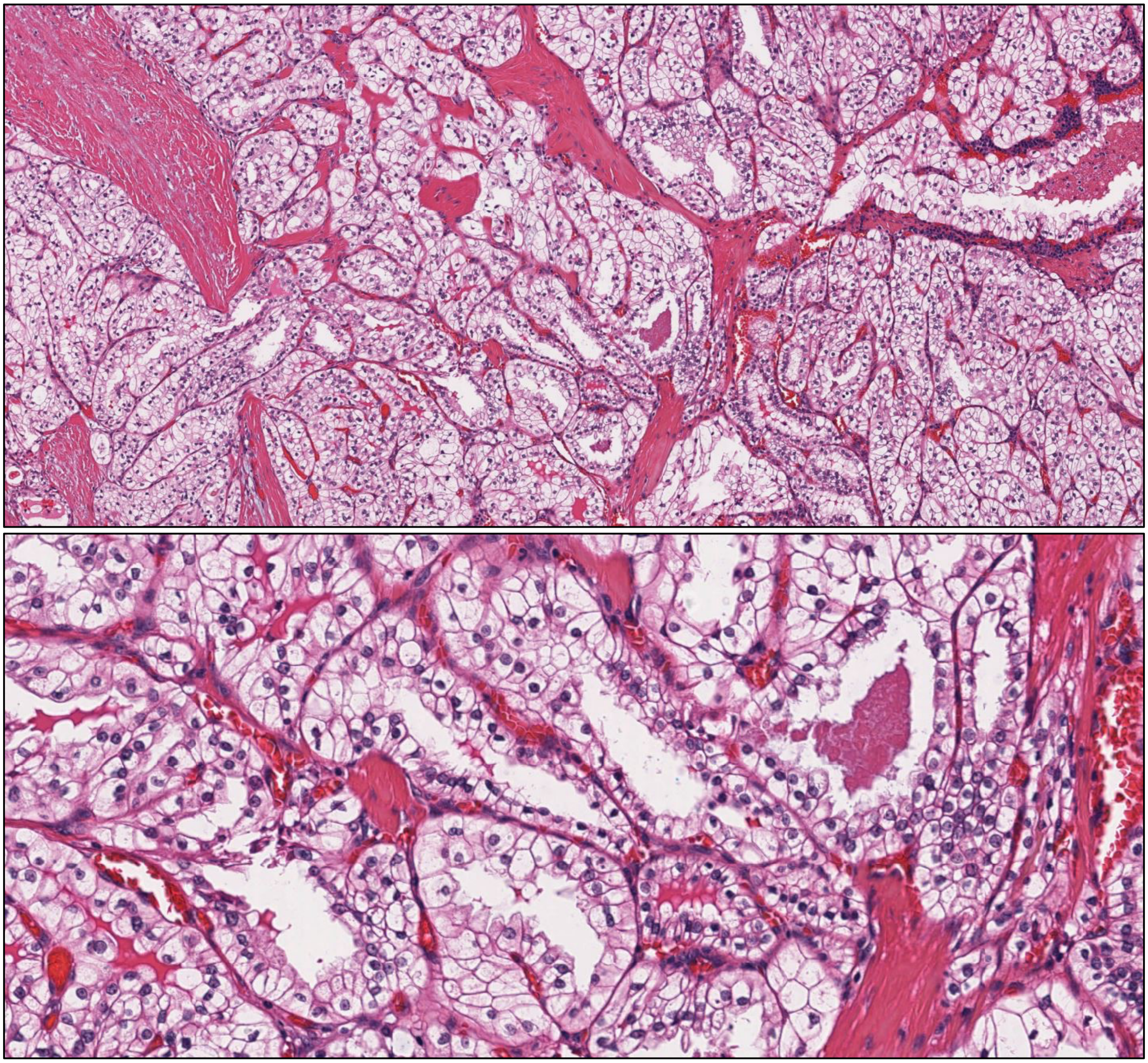

Patient 6. TCGA-DV-A4VZ

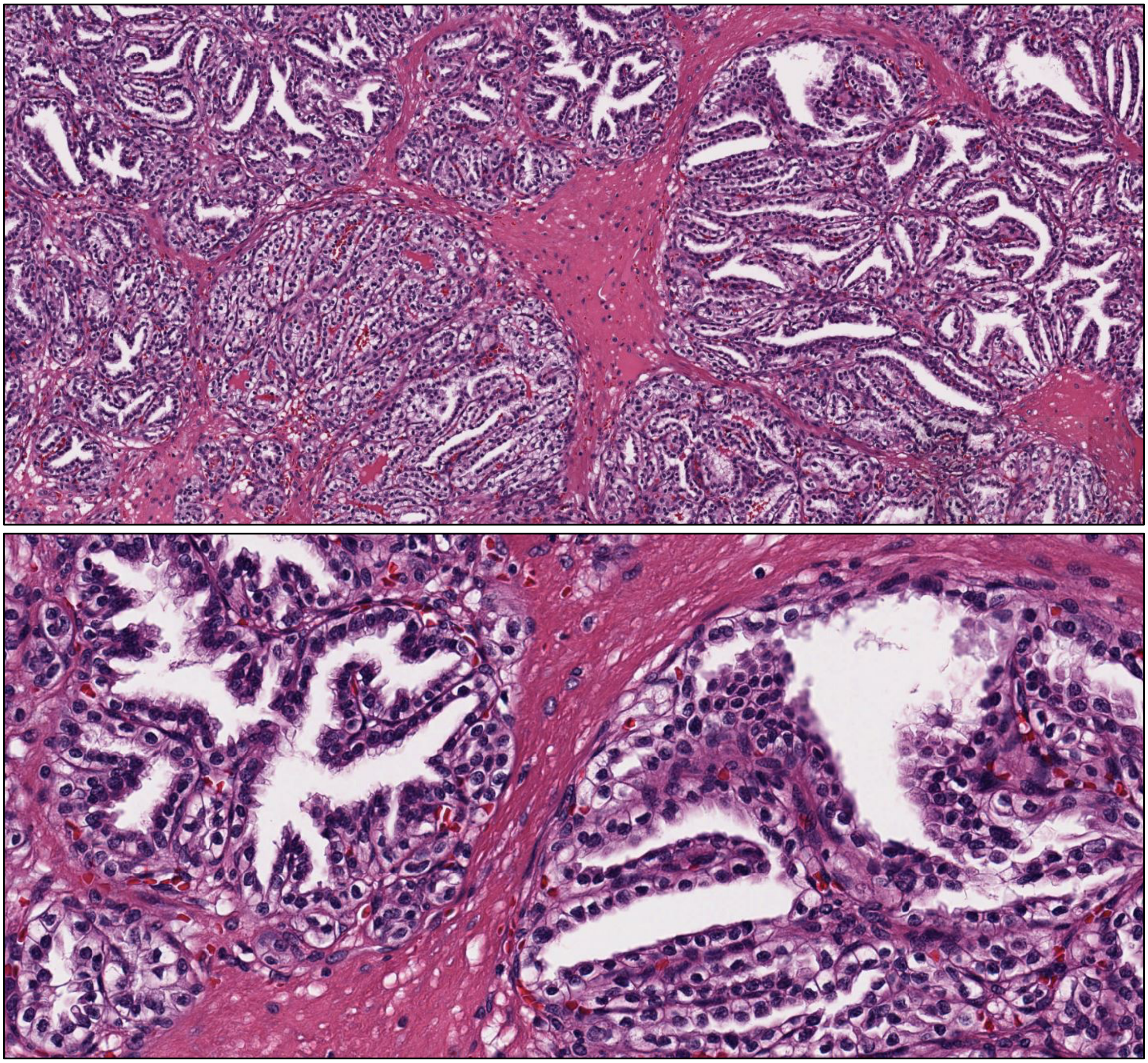

Patient 7. TCGA-DV-A4W0

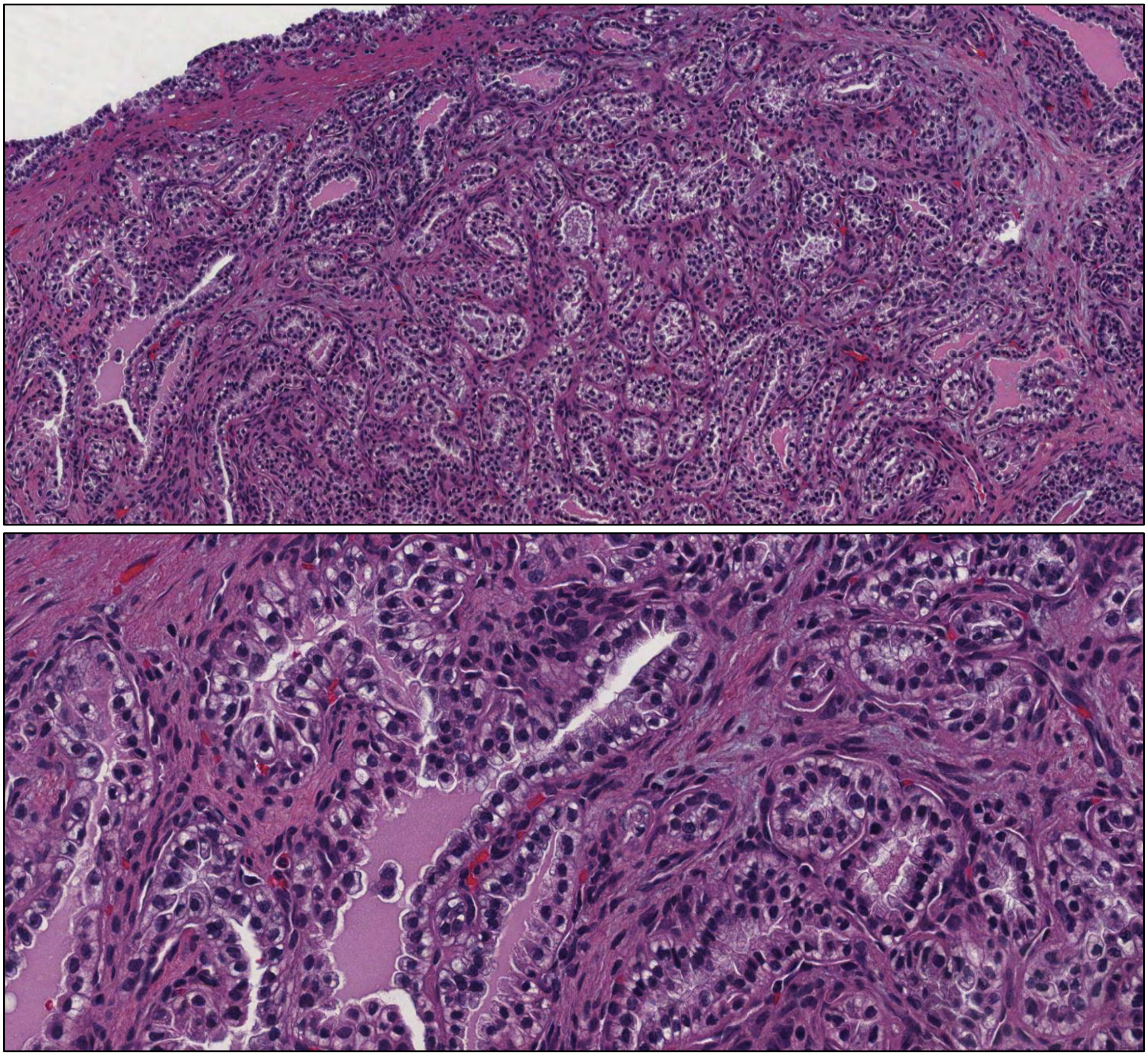

Patient 8. TCGA-DV-5576

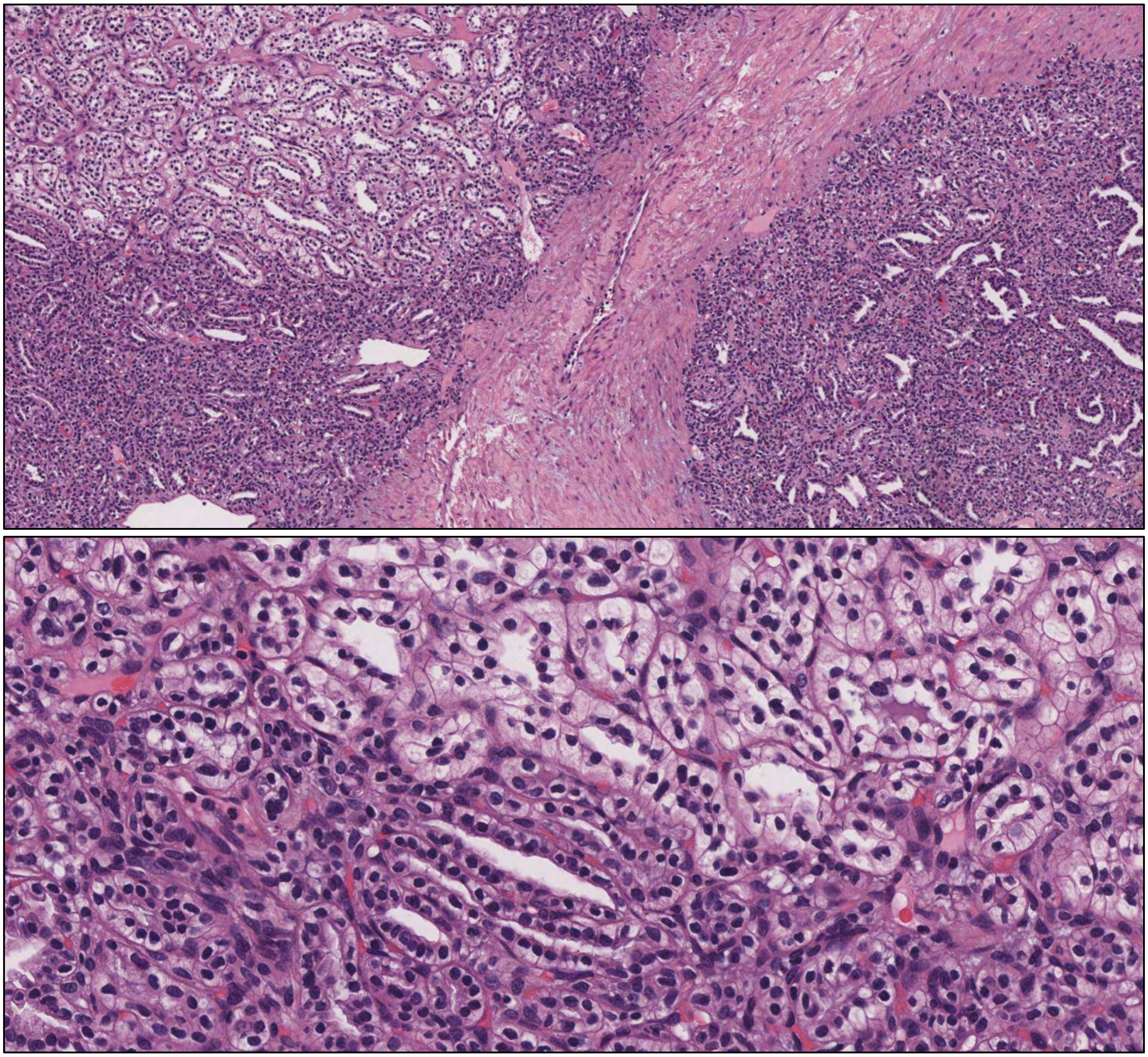

Patient 9. TCGA-2Z-A9J9

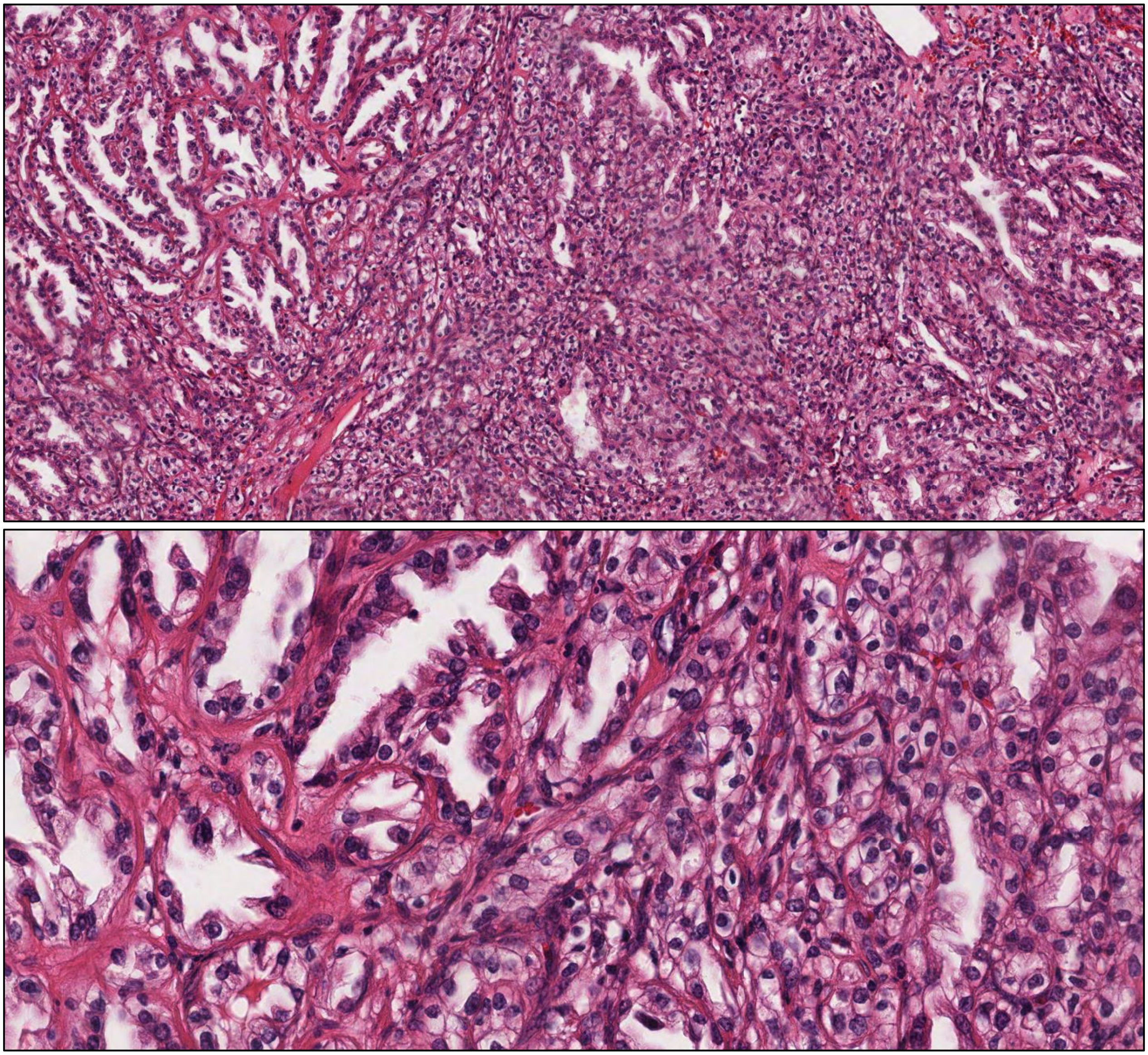

Patient 10. TCGA-A4-7828
